# The human liver microenvironment shapes the homing and function of CD4^+^ T-cell populations

**DOI:** 10.1136/gutjnl-2020-323771

**Published:** 2021-09-21

**Authors:** Benjamin G Wiggins, Laura J Pallett, Xiaoyan Li, Scott P Davies, Oliver E Amin, Upkar S Gill, Stephanie Kucykowicz, Arzoo M Patel, Konstantinos Aliazis, Yuxin S Liu, Gary M Reynolds, Brian R Davidson, Amir Gander, Tu Vinh Luong, Gideon M Hirschfield, Patrick T F Kennedy, Yuehua Huang, Mala K Maini, Zania Stamataki

**Affiliations:** 1 Institute of Immunology and Immunotherapy, University of Birmingham, Birmingham, UK; 2 Institute of Immunity and Transplantation, Division of Infection and Immunity, University College London, London, UK; 3 Centre for Liver and Gastrointestinal Research, University of Birmingham, Birmingham, UK; 4 Department of Infectious Diseases and Guangdong Provincial Key Laboratory of Liver Disease Research, Third Affiliated Hospital of Sun Yat-Sen University, Guangzhou, China; 5 Immunobiology, Blizard Institute, London, UK; 6 Surgery, Royal Free Campus, UCL Medical School, London, UK; 7 Tissue Access for Patient Benefit, University College London, London, UK; 8 Department of Cellular Pathology, Royal Free Hospital, London, UK; 9 Centre for Liver Research, National Institute for Health Research Biomedical Research Unit, University of Birmingham, Birmingham, UK; 10 Department of Infectious Diseases, Third Affiliated Hospital of Sun Yat-sen University, Guangzhou, Guangdong, China; 11 Guangdong Provincial Key Laboratory of Liver Disease Research, Third Affiliated Hospital of Sun Yat-sen University, Guangzhou, Guangdong, China; 12 Division of Infection and Immunity, Rayne Institute, University College London, London, UK

**Keywords:** T lymphocytes, hepatitis B, immunology, liver immunology, cellular immunity

## Abstract

**Objective:**

Tissue-resident memory T cells (T_RM_) are vital immune sentinels that provide protective immunity. While hepatic CD8^+^ T_RM_ have been well described, little is known about the location, phenotype and function of CD4^+^ T_RM_.

**Design:**

We used multiparametric flow cytometry, histological assessment and novel human tissue coculture systems to interrogate the ex vivo phenotype, function and generation of the intrahepatic CD4^+^ T-cell compartment. We also used leukocytes isolated from human leukocyte antigen (HLA)-disparate liver allografts to assess long-term retention.

**Results:**

Hepatic CD4^+^ T cells were delineated into three distinct populations based on CD69 expression: CD69^−^, CD69^INT^ and CD69^HI^. CD69^HI^CD4^+^ cells were identified as tissue-resident CD4^+^ T cells on the basis of their exclusion from the circulation, phenotypical profile (CXCR6^+^CD49a^+^S1PR1^−^PD-1^+^) and long-term persistence within the pool of donor-derived leukcoocytes in HLA-disparate liver allografts. CD69^HI^CD4^+^ T cells produced robust type 1 polyfunctional cytokine responses on stimulation. Conversely, CD69^INT^CD4^+^ T cells represented a more heterogenous population containing cells with a more activated phenotype, a distinct chemokine receptor profile (CX_3_CR1^+^CXCR3^+^CXCR1^+^) and a bias towards interleukin-4 production. While CD69^INT^CD4^+^ T cells could be found in the circulation and lymph nodes, these cells also formed part of the long-term resident pool, persisting in HLA-mismatched allografts. Notably, frequencies of CD69^INT^CD4^+^ T cells correlated with necroinflammatory scores in chronic hepatitis B infection. Finally, we demonstrated that interaction with hepatic epithelia was sufficient to generate CD69^INT^CD4^+^ T cells, while additional signals from the liver microenvironment were required to generate liver-resident CD69^HI^CD4^+^ T cells.

**Conclusions:**

High and intermediate CD69 expressions mark human hepatic CD4^+^ T_RM_ and a novel functionally distinct recirculating population, respectively, both shaped by the liver microenvironment to achieve diverse immunosurveillance.

**Significance of this study**

**What is already known on this subject?**

- Tissue-resident memory CD4^+^ and CD8^+^ T cells are important front-line immune sentinels in many human tissues.
- The human liver has been shown to contain long-lived tissue-resident CD8^+^ T cells that are capable of rapid effector function.
- Liver-resident CD4^+^ T cells remain uncharacterised, and their contribution to health and disease has not yet been studied.

**What are the new findings?**

- CD69 expression identifies three phenotypically and functionally distinct intrahepatic CD4^+^ T-cell populations: CD69^−^CD4^+^, CD69^INT^CD4^+^ and CD69^HI^CD4^+^.
- CD69^HI^CD4^+^ T cells represent a long-lived liver-resident population that expresses classical retention markers, occupies sinusoidal and periportal niches, and is maintained in a resting and restrained state.
- CD69^INT^ marks a population containing both resident and recirculating T cells with differential chemokine and activation profiles.
- CD69^HI^CD4^+^ T cells produce robust T_H_1 cytokine responses, while CD69^INT^CD4^+^T-cells cells favour the production of interleukin-4 on short-term T-cell receptor engagement.
- The frequency of CD69^INT^CD4^+^ T cells correlates with necroinflammatory scores in patients with chronic hepatitis B infection.
- Novel autologous liver slice coculture models promote the differentiation of both CD69^INT^CD4^+^ and CD69^HI^CD4^+^ cells from blood, but hepatic epithelia were sufficient to induce the CD69^INT^CD4^+^ phenotype.

**Significance of this study**

**How might it impact on clinical practice in the foreseeable future?**

- Our study identifies distinct intrahepatic CD4^+^ T cells not detectable in the blood, underscoring the need for continued sampling of the liver.
- An understanding of the differential functionality of CD69^HI^CD4^+^ and CD69^INT^CD4^+^ T cells compartmentalised at the site of pathology has important implications for current intensive efforts to develop immunotherapies for liver diseases.
- The capacity of liver-derived signals to allow *in vitro* recapitulation of tissue-resident CD4^+^ T cells could be exploited for therapeutic targeting.

## Introduction

Tissue-resident memory T cells (T_RM_) are a non-recirculating population that are critical in front-line adaptive immunity. Strategically positioned within tissues, these cells react to pathogen re-exposure more efficiently than circulating memory subsets.^1^ This function is mediated directly and by employing an innate-like ‘sensing and alarm’ strategy to enable recruitment and activation of other effector cells.^2 3^ Human T_RM_ have now been identified in many organs^1 4 5^ and differ substantially from their circulating counterparts in phenotype,^6^ function,^7 8^ metabolism,^9 10^ maintenance requirements^11^ and responsiveness to stimuli.^12^ Expression of tissue retention molecules CD69, CD103 and CD49a and a lack of tissue egress markers including CCR7 and sphingosine-1-phosphate receptor 1 (S1PR1) define T_RM_.^13^ Of these, CD69 is particularly important as a marker preserved on CD4^+^ and CD8^+^ T_RM_ in all tissues,^14^ and separation through expression of this molecule alone has recently been used to define a human T_RM_ transcriptome with strong fidelity to more established murine T_RM_ profiles.^13 15^

Recently, Pallett *et al* identified intrahepatic CD8^+^ T_RM_ (CD69^+^CD103^+^CXCR6^+^CXCR3^+^PD-1^+^ (PD-1 – programmed cell death protein-1)), capable of robust interleukin (IL)-2 production, associated with viral control in the liver of HBV-infected individuals.^5^ However, little is known about CD4^+^ T_RM_ and how the liver shapes their biology. In one study, Wong *et al* outlined distinct activation, differentiation and homing receptor profiles of liver perfusate CD4^+^ T cells as part of a multiorgan mapping study,^16^ supporting the possibility of a liver-resident CD4^+^ T-cell population.

Here, we provide the first comprehensive phenotypical and functional analysis of intrahepatic CD4^+^ T_RM_ in the human liver. We identified two distinct populations of CD69-expressing intrahepatic CD4^+^ T cells: CD69^HI^ and CD69^INT^. CD69^HI^CD4^+^ T cells within the human liver had prototypical hallmarks of tissue residency, including high expression of retention markers, exclusion from the circulation and rapid multifunctional type 1 cytokine production on stimulation. We also report a novel population of intrahepatic CD69^INT^CD4^+^ T cells characterised by a unique chemokine receptor profile (CD69^INT^CX_3_CR1^+^CXCR3^+^CXCR1^+^). CD69^INT^CD4^+^ cells retained the ability to recirculate and on stimulation produced the T_H_2 cytokine IL-4. The frequency of these CD69^INT^CD4^+^ T cells also correlated with necroinflammatory scores in patients with chronic hepatitis B. Finally, we demonstrated that contact with hepatic epithelia drives the CD69^INT^CD4^+^ phenotype, while CD69^HI^CD4^+^ cells required additional signals from the liver microenvironment.

## Materials and methods

### Patient samples and immune cell isolation

Blood, liver and lymph node (LN) samples were obtained from centre A, the Queen Elizabeth Hospital, Birmingham (references 06/Q2702/61 and 06/Q2708/11). Blood, liver (resections, biopsies, fine needle aspirates, HLA-mismatched explants), gut, spleen and LN samples from centre B were obtained from either the Royal Free Hospital, London (references 16/WA/0289, 11/WA/0077, 11/H0720/4 (RIPCOLT clinical trial number 8191) or 11/LO/0421) or Royal London Hospital, Barts Health NHS Trust (references P/01/023, 16/LO/1699 or 17/LO0266). Immune cells were isolated from tissues/blood through tissue digestion and density centrifugation (see online supplemental experimental methods). See online supplemental table 1 for full patient details.

10.1136/gutjnl-2020-323771.supp11 Supplementary data

### Flow cytometry

For surface staining, cells were incubated with fluorescence-conjugated antibodies on ice for 20–30 min. For intracellular staining, cells were either fixed with 1% formaldehyde (Sigma-Aldrich) for 15 min, permeabilised with 0.1% Saponin (Sigma-Aldrich) and stained with relevant antibodies in 0.1% saponin (30 min, 20°C), or fixed and permeabilised with Cytofix/Cytoperm (BD Bioscience) or FoxP3 Buffer Set (BD Bioscience) according to the manufacturer’s instructions, and stained in 0.1% saponin. Dead intrahepatic lymphocytes (IHLs) were identified and excluded using either a fixable live/dead dye (Thermo Fisher) for all centre B samples or zombie dyes (Biolegend) for all cultured centre A samples. Samples were analysed on an ADP CyAn flow cytometer running Summit software (Beckmann Coulter, centre A) or LSRII or X20 flow cytometers running FACSDiva software (BD Bioscience) for samples from centre B (see online supplemental table 2 for the list of antibodies used and online supplemental figure 1 for gating strategies). CD69^−^ and CD69^INT^ populations were distinguished using isotype-matched controls, in combination with peripheral blood staining to determine CD69^INT^ versus CD69^HI^ gate positions.

10.1136/gutjnl-2020-323771.supp1 Supplementary data

### Immunofluorescence

Formalin-fixed paraffin-embedded 3 µm liver sections were deparaffinised with xylene, rehydrated with 99% industrial denatured alcohol and underwent antigen retrieval by microwaving in Tris-based antigen-unmasking solution (Vector Labs). Slides were washed with TBS+0.1% Tween (TBST) and 2× casein solution (Vector labs) was added for 10 min, before 1-hour incubation with primary antibodies diluted in TBST. For antibodies used, see online supplemental experimental procedures. Following three washes with TBST, secondary antibodies were applied for 1 hour in TBST; autofluorescence was quenched with the TrueVIEW autofluorescence quenching kit (Vector Labs); and tissues were mounted with VECTASHIELD Vibrance Antifade Mounting Medium with DAPI (4′,6-diamidino-2-phenylindole, Vector Labs). Tissues were imaged using the Zeiss LSM 880 confocal microscope (Carl Zeiss) equipped with a ×63 water immersion objective.

### T-cell stimulation for assessment of cytokine production

Peripheral blood mononuclear cells (PBMCs) and IHLs were first stained for surface antigens then cultured alone, with 1:1 ratio of anti-CD3/CD28 beads (Dynabeads, ThermoFisher), or 50 ng/mL phorbol 12-myristate 13-acetate (PMA) and 1 µM ionomycin (both Sigma Aldrich, UK), all with 10 µg/mL Brefeldin A (Sigma Aldrich). For culture and media details, see online supplemental experimental procedures.

### CD4^+^ T-cell isolation and cell culture

CD4^+^ T cells were isolated from PBMCs with the EasySep human CD4^+^ T-cell enrichment kit, or EasySep naïve/memory human CD4^+^ T-cell enrichment kits (all StemCell Technologies). T cells/PBMCs were cultured with hepatic epithelial cell lines (Huh-7, HepG2 and Hep3B), hepatic stellate cell line LX-2, primary hepatic sinusoidal endothelial cells (HSECs) and primary biliary epithelial cells (BECs). Primary BEC and HSEC were isolated in-house as previously described.^17 18^ For media details, see online supplemental experimental procedures. 1×10^6^ PBMCs/T-cells were added per well and cultured for up to 7 days. For transwell separation experiments, T cells were added to the top of the 0.4 µm pore transwell insert, separated from hepatic cells at the bottom of the 24-well plate.

### Liver slice cultures

Precision-cut liver slices of 2 mm were prepared using a TruSlice tissue slicer (CellPath) and were cultured in complete Dulbecco's Modified Eagle Medium (DMEM) with 2% foetal bovine serum (FBS) in 48-well plates. Autologous total PBMCs were added in T-cell media (1×10^6^/well), and plates were cultured for 5 hours at 37°C before PBMC harvest and were used in downstream assays.

### Data analysis and statistics

All flow cytometry data were analysed using FlowJo V.9–10 (FlowJo LLC). Statistical testing was applied in Prism V.8 (GraphPad). Median average values and and non-parametric testing were used throughout.

## Results

### CD69 expression distinguishes three intrahepatic CD4^+^ T-cell populations with differential homing potentials

**Figure 1:**
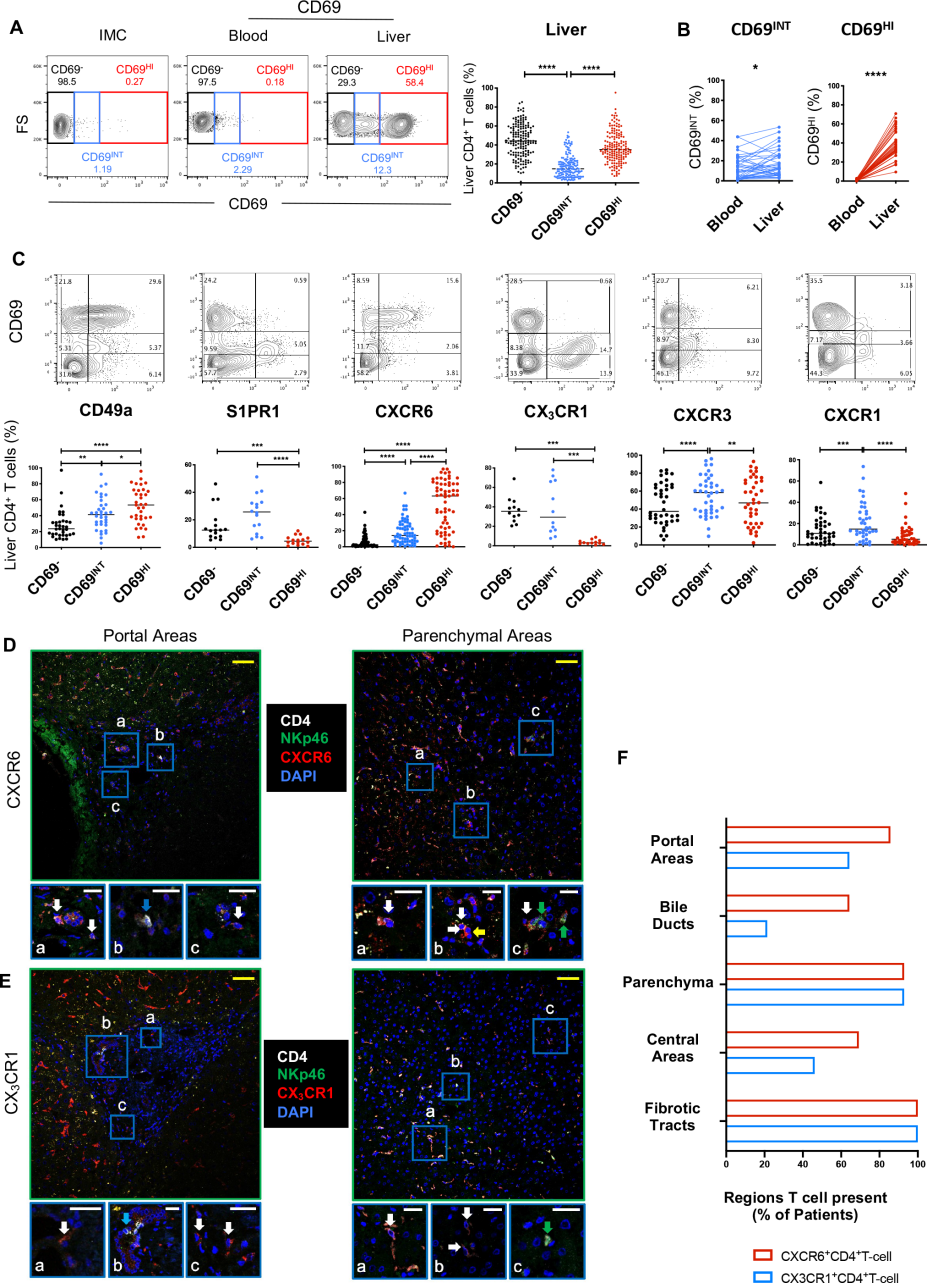
CD69 expression distinguishes three intrahepatic CD4^+^ T-cell populations with differential homing potentials. (A) Gating strategy showing CD69^−^, CD69^INT^ and CD69^HI^ populations. Representative flow cytometry plot for CD4^+^ T-cell distribution in blood and liver, and summary data showing % CD4^+^ T cells in IHL from two independent centres (n=162). Isotype-matched controls were used to set CD69^−^ gates. (B) % CD69-expressing T-cell populations in paired blood and liver (n=39). (C) Expression of key homing and retention markers on CD69-expressing CD4^+^ T cells (% of total CD4^+^ T cells). Images depicting localisation of CXCR6^+^ CD4^+^ T cells (D) or CX_3_CR1^+^ CD4^+^ T cells (E) in portal and parenchymal areas of human livers (representative of n=14 livers (5 control, 4 patients with HBV and 5 patients with PBC)). Sections stained for CD4, NKp46, DAPI and chemokine receptor indicated. Cells of interest expressed both the chemokine receptor and CD4 and lacked NK cell marker NKp46. Areas of interest (A–C) shown at higher magnification below each main image. White arrows: cell of interest, green arrows: NKp46^+^ cell, yellow arrow: chemokine receptor^+^ CD4^−^ cell, blue arrows: CD4^+^ NKp46^-^ CXCR6^-^ cells (D) or CD4+NKp46-CX3CR1- cells (E). Yellow scale bars: 50 µm, white scale bars: 20 µm. (F) Cumulative scoring of the presence of each cell of interest within different liver regions. Cells of interest were scored as present in specific areas if at least three cells were present within each region. Plot shows the % of each region that contained cells of interest (n=14, as above; fibrotic tracts in non-control livers only, n=9). Cells were classed as present in portal regions and central regions if they were identified within 50 µm of their respective vasculature. Association with bile ducts was scored if cells were making direct contact. Statistical comparisons by Freidman tests with Dunn’s multiple tests (A, C); Wilcoxon matched-pair, signed-rank tests (B). p < 0.05 (*), < 0.01 (**), < 0.001 (***), < 0.0001 (****) FS, forward scatter; IHL, intrahepatic lymphocyte; IMC, isotype-matched control; NK, natural killer; PBC, primary biliary cholangitis.

To identify intrahepatic CD4^+^ T_RM_, we analysed CD69 expression in over 160 liver samples from two research centres. Three intrahepatic CD4^+^ T-cell phenotypes were identified: CD69^−^, CD69^INT^ and CD69^HI^ (figure 1A). CD69^HI^CD4^+^ cells were negligible in blood, while CD69^INT^CD4^+^ T cells were detected in both intrahepatic and peripheral pools (figure 1B). In the liver, CD69^HI^CD4^+^ T cells displayed striking concordance with a residency-associated profile (CD49a^+^CXCR6^+^S1PR1^−^CX_3_CR1^−^) (figure 1C). By contrast, CD69^INT^CD4^+^ T cells retained expression of the tissue egress marker S1PR1 and fractalkine receptor CX_3_CR1, which is associated with migratory T cells,^13 19 20^ as well as the strongest expression of parenchymal homing receptors CXCR3 and CXCR1.^21^ Hepatic CD69^INT^CD4^+^ T cells expressed less CD49a and CXCR6 than CD69^HI^CD4^+^ T cells, although these residence markers were all expressed to a higher extent than on the CD69^−^CD4^+^ T cells.

In keeping with their association with residence, fine-needle aspirate (FNA) samples (that sample more blood-derived than interstitial T cells compared with biopsies^22^) showed a more marked reduction in the frequency of CD69^HI^CD4^+^ than CD69^INT^CD4^+^ T cells compared with matched liver tissue obtained by biopsy (online supplemental figure 2A). Similarly, of the three populations, only CD69^HI^CD4^+^ T cells were enriched for an effector memory phenotype—a prerequisite for T_RM_ cells (online supplemental figure 2B). Interestingly, more CD69^INT^CD4^+^ T cells expressed a gut homing signature (CCR9, integrin α4β7) than the other two CD69-expressing populations, suggestive of a potential wider enteric surveillance role (online supplemental figure 2C). Additional profiling revealed increased CCR5 expression on CD69^HI^CD4^+^ T cells, higher expression of CCR6 on both CD69-expressing populations than CD69^−^CD4^+^ T cells and no differential CCR10 expression. The retention marker CD103 was expressed most on CD69^HI^CD4^+^ T cells, although this frequency was low, as reported for other human resident CD4^+^ T-cell subsets (online supplemental figure 2C).^4^ Together, our data reveal two distinct CD69-expressing CD4^+^ T-cell populations in the human liver: CD69^HI^CD4^+^ T cells with the strongest T_RM_ profile and CD69^INT^CD4^+^ T cells with differential homing potential.

10.1136/gutjnl-2020-323771.supp2 Supplementary data

Next, we assessed whether differential expression of CXCR6 and CX_3_CR1 between the different intrahepatic CD4^+^ T-cell populations affected their hepatic distribution (online supplemental figure 3A). CD4^+^ T cells expressing either chemokine receptor were found throughout the liver—in both portal and central areas, in fibrotic tracts and throughout the parenchyma where they likely play a crucial role in the immunosurveillance of hepatocytes (figure 1D, E, and online supplemental figure 3A–C). CXCR6^+^CD4^+^ T cells (enriched for the CD69^HI^CD4^+^ T-cell population) were found more frequently in association with bile ducts (figure 1F), in keeping with the role of this receptor in biliary homing.^23^

10.1136/gutjnl-2020-323771.supp3 Supplementary data

### High CD69 expression marks a CD4^+^ T-cell population capable of long-term residence within the liver

**Figure 2:**
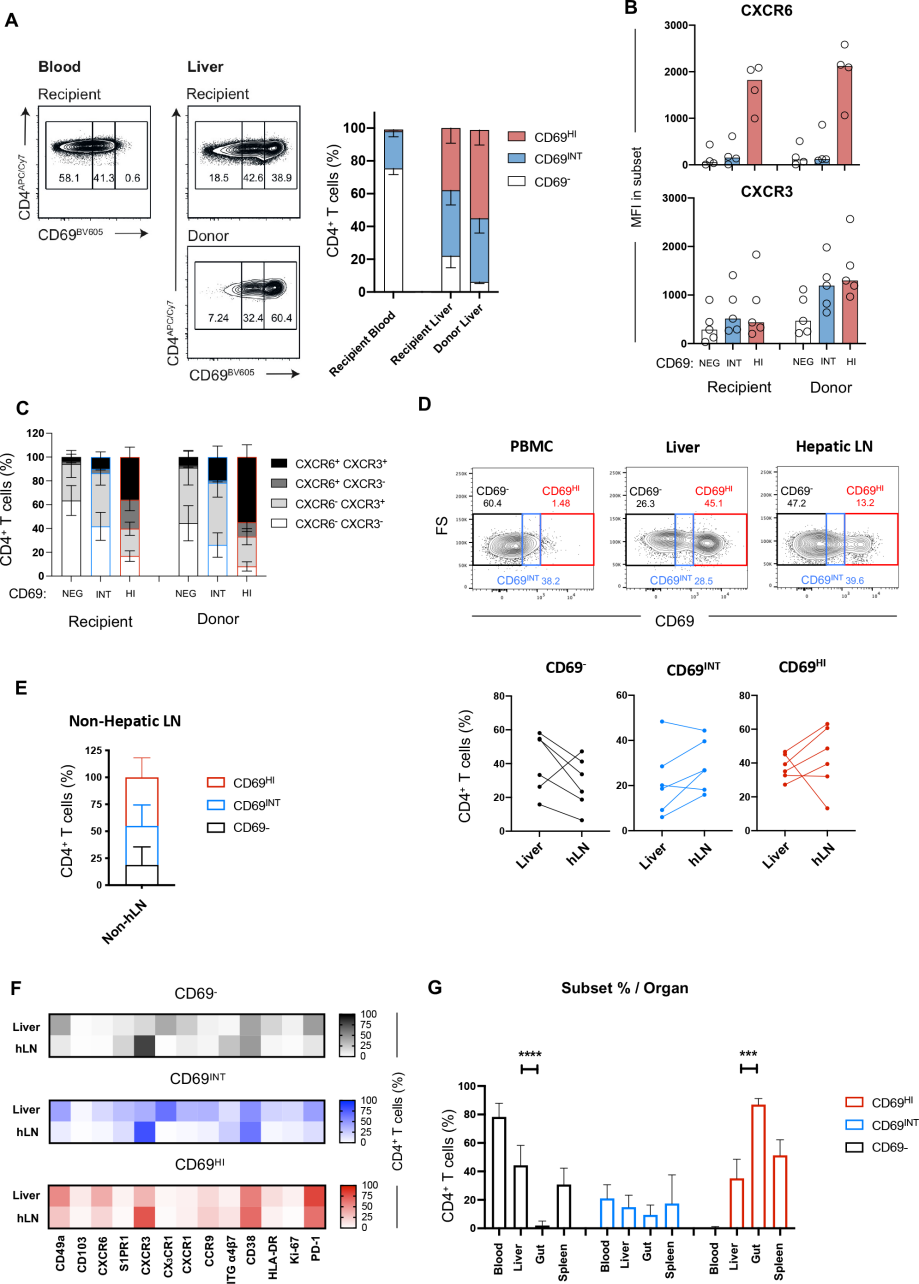
High CD69 expression marks a CD4^+^ T-cell population capable of long-term residence within the liver. (A) HLA-mismatched allograft sampling allows assessment of resident T cells. Donor-derived T cells are distinguished from recipient-derived T cells through HLA staining. Example distributions of CD69^−^, CD69^INT^ and CD69^HI^ cells in recipient and donor pools of liver and blood samples, and combined data across five patient samples. (B) MFI of CXCR6 and CXCR3 expressions in the three populations in donor and recipient pools. (C) Breakdown of CXCR3/CXCR6 coexpression patterns in different donor and recipient subpopulations (n=4). (D) Staining and combined data showing population distribution from liver (n=6), hepatic LNs (n=6) and non-hepatic (mesenteric) LNs (n=6). Example plots show hLN, PBMC and liver as gating controls. (E) Subset breakdown in distal non-hLNs. (F) Heatmap of % marker expression in CD69^−^ (top), CD69^INT^ (middle) and CD69^HI^ (bottom) from matched liver and hLN samples. CD103, n=6; CD49a, n=5; CXCR6 and HLA-DR, n=4; CXCR3, CXCR1, PD-1 and CD38, n=3; S1PR1, CCR9, integrin α4β7 and Ki-67, n=2; CX_3_CR1, n=1. (G) Frequency of CD69^−^, CD69^INT^ and CD69^HI^ CD4^+^ T cells in blood (n=103), liver (n=118), gut (n=6) and spleen (n=4) samples. Statistical comparisons on paired populations by Wilcoxon matched-pair, signed-rank tests (A–D, F), and Kruskal-Wallis tests with duns post hoc tests on liver, gut and spleen samples within each CD4^+^ T-cell subset (G). HI, high; hLN, hepatic lymph node; INT, intermediate; LN, lymph node; MFI, Median fluorescence intensity, NEG, negative.

To ascertain which population was strictly resident in the human liver, we examined HLA-mismatched allograft samples explanted up to a decade after initial transplantation.^24^ In our recent study, we showed that in all cases, a small pool of long-lived, donor-derived CD4^+^ T cells were detected by staining with HLA-specific antibodies.^24^ No donor-derived CD4^+^ T cells were detected in the blood, confirming that donor-derived cells and their progeny were maintained locally in the liver allograft.^24^ Re-examining the CD4^+^ T-cell fraction from donor and recipient pools, we observed that CD69^HI^CD4^+^ T cells were significantly enriched in the persisting, donor-derived fraction, establishing these cells as T_RM_ (figure 2A). By contrast, CD69^−^CD4^+^ T cells comprised a negligible fraction of the long-lived donor-derived T cells. Interestingly, however, a population of CD69^INT^CD4^+^ T cells was detected in the donor-derived compartment in all cases, suggestive of the long-term retention of some of these cells. Examining the recipient-derived CD4^+^ T cells infiltrating the allograft, we found these to be capable of acquiring both a CD69^HI^CXCR6^HI^ and a CD69^INT^CXCR6^LO^ phenotype, suggesting that infiltrating T cells are shaped by the hepatic microenvironment (figure 2B). By contrast, recipient CD4^+^ T cells within the allograft only showed a subtle increase in CXCR3 compared with their circulating counterparts, expressing much less than the donor-derived CD69^HI^CD4^+^ and CD69^INT^CD4^+^ T cells, suggesting CXCR3 is less easily imprinted on liver infiltration (figure 2B). Donor-derived CD69^HI^CD4^+^ T cells contained a greater representation of dual CXCR6^+^CXCR3^+^-expressing cells than their recipient-derived counterparts, while the CXCR6^−^CXCR3^+^ was most enriched on the donor-derived CD69^INT^CD4^+^ T-cell population (figure 2C). This suggests expression of both markers is important in long-term retention of CD69^HI^CD4^+^ T cells, with CD69^INT^CD4^+^ T cells more reliant on CXCR3 alone.

Reasoning that expression of tissue egress markers S1PR1 and CX_3_CR1 would imbue CD69^INT^CD4^+^ T cells with the ability to recirculate through lymphatics, we assessed the make-up of matched liver and liver-draining hepatic hilar LN (hepatic lymph node (hLN), figure 2D) and distal non-hLN (figure 2E). Both CD69^INT^CD4^+^ and CD69^HI^CD4^+^ T cells were present in LNs, supporting this possibility. While donor-matched liver and hLN CD69^HI^CD4^+^ T cells were phenotypically similar, reflective of a common residency signature, CD69^INT^CD4^+^ and CD69^−^CD4^+^ cells in hLNs differed substantially from their liver equivalents in their chemokine receptor profile (subsets in hLNs enriched for CXCR3, but depleted for CX_3_CR1, CXCR1 and CCR9 expression; figure 2F). Furthermore, CD69^HI^CD4^+^ and CD69^INT^CD4^+^ were detectable not only in the liver and LNs but also in the gut, where CD69^HI^CD4^+^ predominates, and in spleen samples (figure 2G).

Thus, while the properties of long-lived tissue enrichment and absence from the peripheral circulation mean CD69^HI^CD4^+^ T cells comply with a tissue-resident definition, CD69^INT^CD4^+^ T cells may represent a population with a context-dependent capacity for liver occupancy and egress.

### CD69^HI^CD4^+^ T_RM_ demonstrates a restrained, resting phenotype, while CD69^INT^CD4^+^ T cells exhibit features of activation

**Figure 3:**
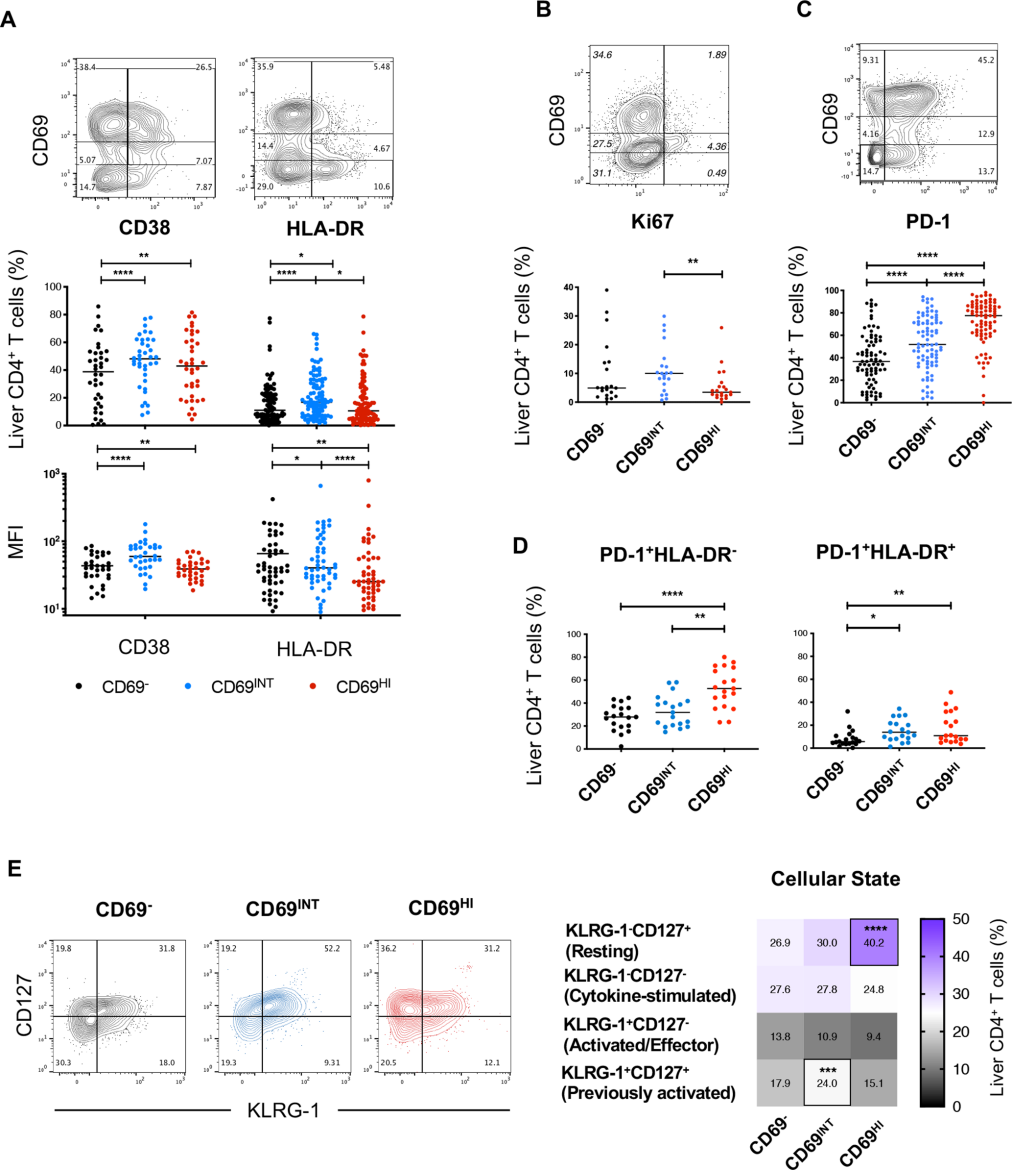
CD69^HI^CD4^+^ T_RM_ demonstrate a restrained, resting phenotype, while CD69^INT^CD4^+^ T cells exhibit features of activation. (A) % and MFI expression of CD38 and HLA-DR. % expression of (B) Ki-67 and (C) PD-1 expressions among the three CD4^+^ T-cell populations. (D) Subset representation among PD-1^+^HLA-DR^−^ and PD-1^+^HLA-DR^+^ designations (n=19). (E) Analysis of the four differentiation/cellular states based on KLRG-1 and CD127 expressions (n=41). Heatmap shows % expression of each designation. Freidman’s tests with Dunn’s multiple tests were used for statistical analysis (A–E) p < 0.05 (*), < 0.01 (**), < 0.001 (***), < 0.0001 (****). MFI, mean fluorescence intensity.

Alongside a tissue-residence marker, CD69 has been used as an indicator of early lymphocyte activation.^25^ Therefore, we examined the activation status of the three hepatic CD4^+^ T-cell populations. Intriguingly, the extent of cellular activation did not correlate with levels of CD69 expression; CD69^INT^CD4^+^ T cells were enriched for activation markers CD38 and HLA-DR, expressing more CD38 than CD69^−^CD4^+^ T cells, and more HLA-DR than CD69^HI^CD4^+^ T cells (figure 3A). Consistent with recent activation in vivo, the CD69^INT^CD4^+^ T-cell population also expressed more Ki67 than their CD69^HI^CD4^+^ T-cell counterparts (figure 3B). Regulatory T cells (T_REG_, CD4^+^CD25^HI^CD127^LO^) were not significantly enriched in any intrahepatic CD4^+^ population, irrespective of CD69 expression, and T_REG_ functional markers cytotoxic T lymphocyte-associated protein-4 (CTLA4) and CD39 were similarly expressed by both CD69-expressing populations when compared with CD69^−^CD4^+^ T cells (online supplemental figure 4).

10.1136/gutjnl-2020-323771.supp4 Supplementary data

Another hallmark of human T_RM_ is the adoption of a self-restrained, resting state necessary to prevent inflammatory damage to residing tissues.^5 13 15^ Correspondingly, CD69^HI^CD4^+^ T cells were enriched for PD-1, with CD69^−^CD4^+^ T cells displaying the lowest frequency (figure 3C). As PD-1 can also denote activation,^26^ we assessed the coexpression of PD-1 and HLA-DR. The percentage of PD-1^+^HLA-DR^−^ cells were enriched within the CD69^HI^CD4^+^ T-cell population, suggesting PD-1 upregulation in CD69^HI^CD4^+^ T cells was not simply an activation phenomenon (figure 3D). To investigate cellular activation states in more detail, we also analysed coexpression patterns of killer cell lectin-like receptor-G1 (KLRG-1, a marker of antigen experience) and CD127, an indicator of common γ-chain cytokine sensitivity.^27 28^ As in human T_RM_ studies,^29^ CD69^HI^CD4^+^ T cells contained the most resting (KLRG-1^−^CD127^+^) cells, whereas CD69^INT^CD4^+^ T cells were enriched for the previously activated (KLRG-1^+^CD127^+^) population (figure 3E).

These data illustrate differences in activation states between CD69^HI^CD4^+^ and CD69^INT^CD4^+^ T cells, with the former exhibiting a resting/restrained phenotype in keeping with their profile as liver T_RM_, while the latter population displayed features consistent with recent activation.

### Liver CD69^HI^CD4^+^ and CD69^INT^CD4^+^ T cells are skewed towards T_H_1 and T_H_2 functional profiles, respectively

**Figure 4:**
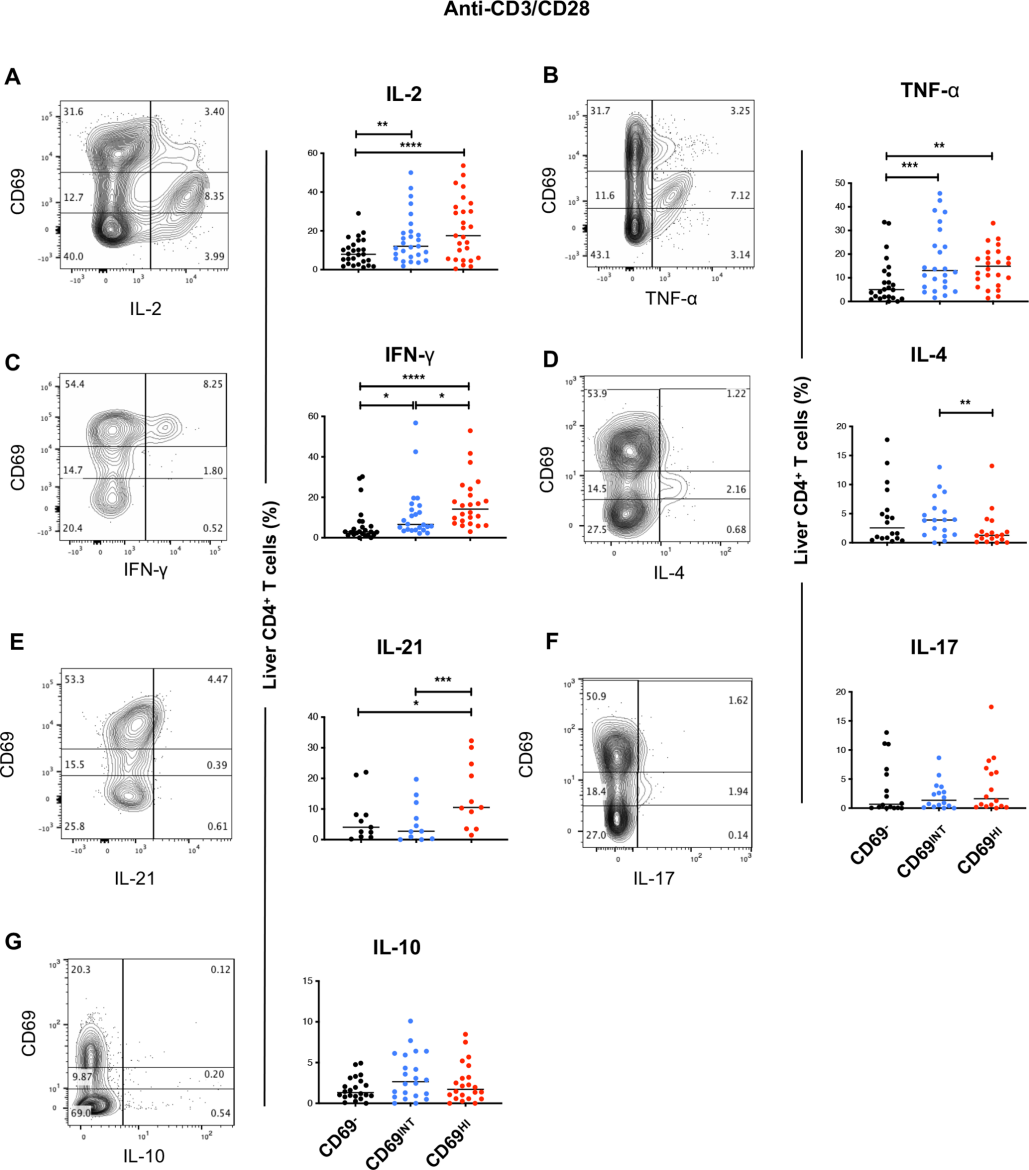
Liver CD69^HI^CD4^+^ and CD69^INT^CD4^+^ T cells are skewed towards T_H_1 and T_H_2 functional profiles, respectively. Each sample was stained with CD69 prior to stimulation to exclude effects of altered CD69 levels due to cellular activation. Representative flow plots and combined data of % expression of six prototypical T_H_ cytokines after 5 hours of stimulation of IHL with anti-CD3/CD28: (A) IL-2 (n=27), (B) TNF-α (n=24), (C) IFN-γ (n=24), (D) IL-4 (n=18), (E) IL-21 (n=11), (F) IL-17 (n=16), (G) IL-10 (n=22). See online supplemental table 4 for disease breakdowns. Freidman’s tests with Dunn’s multiple tests were used for statistical analysis (A–F). IFN-γ, interferon gamma; IHL, intrahepatic lymphocyte; IL, interleukin; TNF-α, tumour necrosis factor alpha.

CD4^+^ T_RM_ cells have a superior functional capacity to circulating T cells and mediate protection against a number of viral infections in multiple organs.^1 15^ To assess the functional potential of CD69^HI^CD4^+^ T_RM_ and CD69^INT^CD4^+^ T cells, intrahepatic leukocytes from a subset of livers were first prestained for CD69 expression to rule out stimulation-induced changes to CD69 expression and then were stimulated to assess their capacity for cytokine production. Following T-cell receptor (TCR) ligation with 5-hour anti-CD3/CD28 stimulation, more CD69^HI^CD4^+^ T cells produced interferon gamma (IFN-γ) and IL-21 than any other population, and both CD69^HI^ and CD69^INT^CD4^+^ T cells were enriched for IL-2, and tumour necrosis factor alpha (TNF-α) compared with their CD69^−^CD4^+^ counterparts (figure 4). Among the two CD69-expressing populations, CD69^INT^CD4^+^ T cells expressed more IL-4, with no differential expression patterns noted for IL-17 or IL-10.

We also assessed the maximum functional capacity of these cells following stimulation with mitogens PMA and ionomycin, and the direct *ex vivo* cytokine levels produced without exogenous stimulation. Similar to anti-CD3/CD28 stimulation, IFN-γ and IL-21 were also highest in CD69^HI^CD4^+^ T cells in PMA/ionomycin-stimulated conditions, and IL-4 was similarly enriched in CD69^INT^ versus CD69^HI^CD4^+^ T cells (online supplemental figure 5A). This stimulation also demonstrated CD69^HI^CD4^+^ T cells possessed the greatest potential to produce IL-2 and TNF-α and a higher potential than CD69^−^CD4^+^ T cells to produce IL-17. Importantly, in the absence of an exogenous stimulation, CD69^INT^CD4^+^ T cells produced IL-4, and CD69^HI^CD4^+^ T cells showed a small enrichment for IFN-γ and IL-21 production compared with CD69^−^CD4^+^T-cells (online supplemental figure 5B), perhaps reflecting recent in vivo stimulation. IL-10 was not enriched in any subset irrespective of stimulation, in line with the lack of an over-representation of a T_REG_ phenotype in either CD4+ T-cell subset (online supplemental figure 4).

10.1136/gutjnl-2020-323771.supp5 Supplementary data

**Figure 5:**
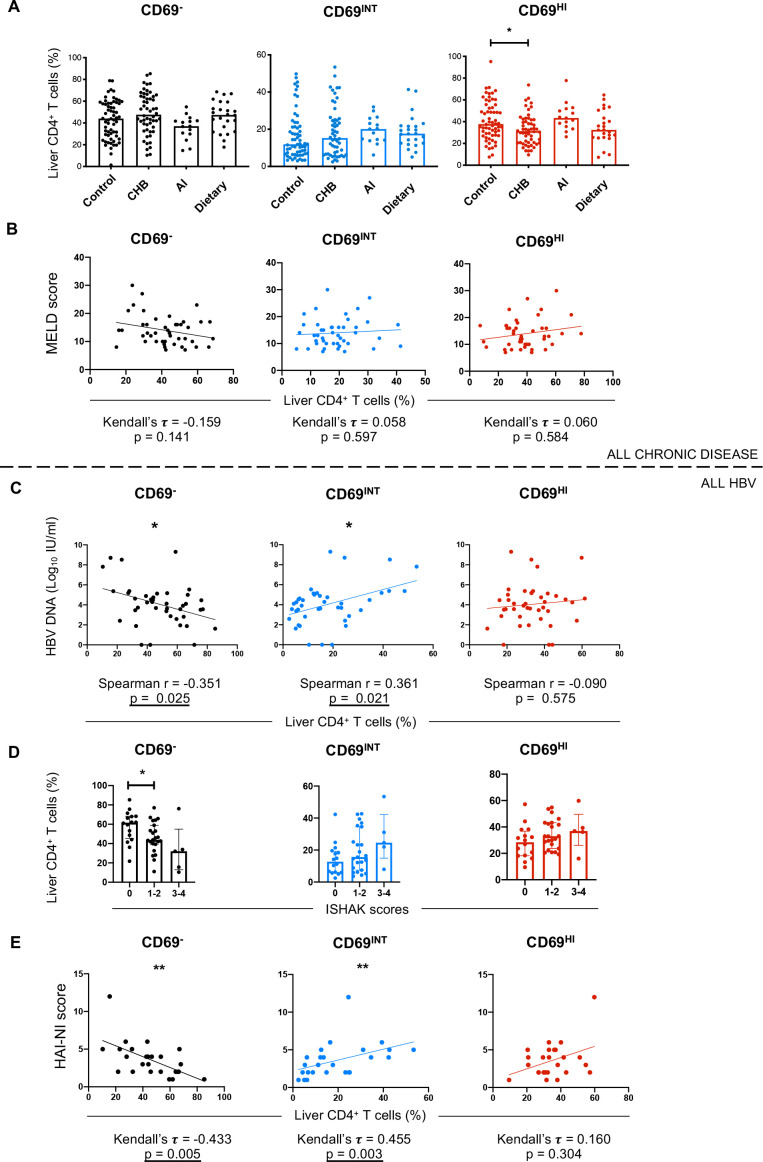
Increased CD69^INT^CD4^+^ T-cell frequencies correlate with necroinflammation in CHB. (A) Representation of each population in control livers (n=62 (11 donor explant transplant rejections, 5 healthy tissue biopsies, 36 colorectal cancer margin liver explants, 8 HCC margin liver explants and 2 cyst-free areas of PLD explants)), patients with chronic HBV (CHB, n=54), autoimmune liver disease (n=15 (6 PBC, 8 PSC and 1 AIH)), and dietary liver disease (n=24 (16 ALD and 8 NASH)). (B) Correlation analysis of patient MELD scores versus % of each of the three subsets for all donors with end-stage liver disease from centre A. (C) HBV DNA, Ishak scoring (D) and HAI-NI scoring (E) plotted against % of each T-cell population in the HBV cohort. Correlation and p values reported for each plot. Statistical testing used: Kruskal-Wallis tests with Dunn’s multiple post hoc tests (A, C), Kendall’s tau rank correlation tests (B, E), Spearman’s rank order correlation (C). AI, autoimmune; AIH, autoimmune hepatitis; ALD, alcoholic liver disease; CHB, chronic HBV infection; HAI-NI, Histology Activity Index-Necroinflammatory; HCC, hepatocellular carcinoma; NASH, non-alcoholic steatohepatitis; PBC, primary biliary cholangitis; PLD, polycystic liver disease; PSC, primary sclerosing cholangitis.

Human T_RM_ from other organs are often polyfunctional, producing the cytokines IFN-γ, TNF-α and IL-2 simultaneously, a property that equips these T cells for better pathogen control.^30–34^ Likewise, we observed an increase in IL-2^+^TNF-α^+^IFN-γ^+^ type 1 multifunctional cells in CD69^HI^CD4^+^ T cells compared with CD69^−^CD4^+^ T cells, a feature not shared by CD69^INT^CD4^+^ T cells (online supplemental figure 5C). Finally, assessment of CD4^+^ T-cell transcription factors revealed an increase in T-bet in CD69^HI^CD4^+^ T cells in line with an enrichment for IFN-γ production, but we were unable to detect T_H_2 transcription factor GATA-3 in hepatic CD4^+^ T cells (online supplemental figure 6). RORγt and FoxP3 were not differentially enriched in either CD69-expressing subset.

10.1136/gutjnl-2020-323771.supp6 Supplementary data

**Figure 6:**
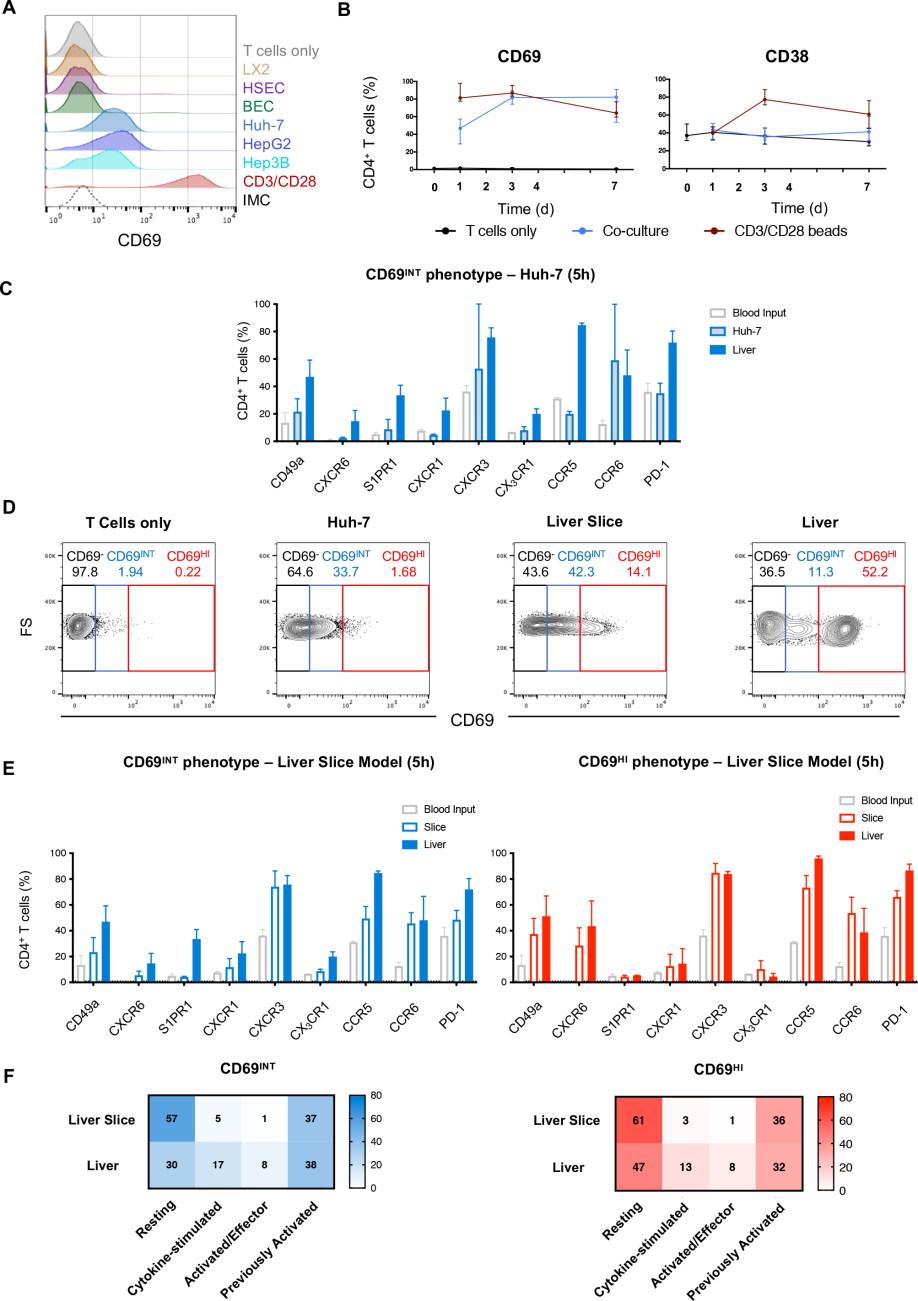
CD69^INT^CD4^+^ and CD69^HI^ CD4^+^ T cells are differentially induced by the liver microenvironment. (A) % CD69 expression on PBMC-derived CD4^+^ T cells cultured for 16 hours with primary HSEC, primary BEC; hepatic stellate cell line LX-2; hepatocyte cell lines HuH-7, HepG2 or Hep3B; with anti-CD3/CD28; or alone. Histogram displays representative CD69 expression levels in each condition. (B) % CD69 and CD38 expressions on blood CD4^+^ T cells over a 7-day culture period with HuH-7 (n=8–10/timepoint). (C) Comparison of key phenotypical markers in Huh-7-generated CD69^INT^ cells from PBMC following 5-hour culture, matched patient IHL CD69^INT^ cells and blood T cells alone (n=2). (D) Representative flow plots showing degree of CD69^INT^ and CD69^HI^ generation within PBMC after 5 hours of culture: alone, with Huh-7 cells, with precision-cut donor-matched liver slices; or from directly isolated IHLs from matched human liver. (E) Comparison of CD69^INT^ cells (left) and CD69^HI^ cells (right) generated from donor-matched PBMCs in a precision-cut liver slice model, with matched donor-derived liver populations, and input blood CD4^+^ T cells alone (n=2). (F) Activation/differentiation statuses of CD69^INT^ and CD69^HI^ cells in the different conditions as assessed by KLRG-1/CD127 costaining patterns (as in figure 3E). Colour intensity and displayed numbers represent median % in each KLRG-1/CD127 designation. BEC, biliary epithelial cell; FS, forward scatter; HL, intrahepatic lymphocyte; HSEC, hepatic sinusoidal endothelial cell; IMC, isotype-matched control.

Taken together, CD69^HI^CD4^+^ and CD69^INT^CD4^+^ T cells are functionally distinct, with CD69^HI^CD4^+^ T cells favouring IFN-γ and type 1 multifunctional responses, while CD69^INT^ expression predisposed CD4^+^ T cells to enhanced IL-4 production.

### Increased CD69^INT^CD4^+^ T-cell frequencies correlate with necroinflammation in chronic HBV infection (CHB)

Next, we stratified liver samples into CHB, autoimmune (autoimmune hepatitis, primary biliary cholangitis and primary sclerosing cholangitis), dietary-induced liver disease (alcoholic liver disease and non-alcoholic steatohepatitis) and control (healthy preimplant, healthy transplant rejections, non-tumour-associated colorectal cancer and hepatocellular carcinoma margins and cyst-free margins of polycystic liver disease tissue) groups to test for population enrichment across diseases. CD69^HI^CD4^+^ T-cell frequencies showed a modest yet consistent reduction in patients with CHB compared with control livers, but no other disease-specific differences were observed (figure 5A). To test if activated CD69^INT^CD4^+^ T cells correlated with disease progression, we analysed model for end-stage liver disease (MELD) scores (a commonly used metric to assess severity of non-viral chronic liver disease^35^) in explanted livers from patients with chronic hepatitis. There was no correlation with either CD4^+^T-cell population and MELD score, irrespective of liver disease aetiology, potentially reflective of a putative role for these cells in both health and disease (figure 5B).

Progression to advanced fibrosis in CHB is highly heterogeneous, with the duration of infection and phase of disease contributing to this process.^36^ When analysing patients with CHB by hepatitis B ‘e’ antigen (HBeAg) seropositivity, viraemia, or the extent of liver inflammation using serum alanine aminotransferase (ALT) concentrations,^36^ we noted that CD69^INT^CD4^+^ T-cell frequencies correlated weakly with serum HBV DNA (figure 5C). The presence of HBeAg or serum ALT did not correlate with any CD4^+^ T-cell population (online supplemental figure 7A–B). Combined analysis of these three metrics into the distinct clinical phases also revealed no subpopulation-linked association (online supplemental figure 7C).

10.1136/gutjnl-2020-323771.supp7 Supplementary data

In order to further assess any links between populations and degree of fibrosis, and ongoing necroinflammatory activity, we subcategorised the patients using the validated Ishak and Histology Activity Index-Necroinflammatory scoring systems.^37^ Frequencies of CD69^INT^CD4^+^ T cells and CD69^HI^CD4^+^ T_RM_ cells did not correlate with the extent of fibrosis by Ishak scoring (figure 5D). However, CD69^INT^CD4^+^ T cells were more frequently observed in patients with a higher intrahepatic necroinflammatory score (figure 5E). Together, these data suggest that activated CD69^INT^ cells may play a role in inflammatory processes of CHB.^38^

### CD69^INT^CD4^+^and CD69^HI^CD4^+^ T-cells are differentially induced by the liver microenvironment

Finally, we sought to determine the origin of these distinct liver CD4^+^ T-cell populations by deconstructing the contribution of different hepatic cell types *in vitro*. To investigate the role of hepatic epithelia, we first cultured PBMC-derived CD4^+^ T cells with different hepatocyte cell lines (Huh-7, HepG2 and Hep3B); within 16 hours, we observed a clear induction of intermediate CD69 expression (figure 6A). By contrast, neither primary HSEC, primary BEC nor the hepatic stellate cell line, LX-2, was able to induce CD69 in the same time frame. However, primary BECs were able to strongly promote the increase in CD69^INT^CD4^+^ T cells from 72 hours, suggesting that sustained interaction with liver epithelia may be necessary for the generation of this population (online supplemental figure 8A–B). Hepatic epithelial-induced CD69 upregulation to an intermediate level was not simply a feature of their activation, as no concomitant upregulation of prototypical T-cell activation marker CD38 was seen, and conventional T cell activation with anti-CD3/CD28 led only to high CD69 expression (figure 6A–B). Mechanistically, this intermediate CD69 induction required direct T cell–epithelial cell contact, and was most efficient in memory CD4^+^ T cells (online supplemental figure 8C–D). *In vitro*-generated CD69^INT^CD4^+^ T cells partially recapitulated the phenotypical signature of intrahepatic CD69^INT^CD4^+^ T cells observed *ex vivo* after just 5 hours in culture, with upregulation of CXCR3 and CD49a, but not S1PR1, CXCR1 or CX_3_CR1 (figure 6C). CD69^INT^CD4^+^ T cells generated *in vitro* were also capable of producing IL-4 on stimulation, again resembling intrahepatic CD69^INT^CD4^+^ T cells (online supplemental figures 4 and 9). Thus, short-term contact with hepatic epithelia can induce a population of CD69^INT^CD4^+^ T cells *in vitro*.

10.1136/gutjnl-2020-323771.supp8 Supplementary data

10.1136/gutjnl-2020-323771.supp9 Supplementary data

We further considered whether additional signals from the liver microenvironment were required to generate CD69^HI^CD4^+^ T cells. To investigate this, we used a coculture model of patient-derived PBMCs with autologous precision-cut liver slices to allow full retention of the native liver microenvironment (figure 6D). Coculture of autologous PBMC for 5 hours with matched liver slices led to an increase in T-cell expression of both intermediate and high levels of CD69, not seen with hepatic epithelia coculture. Remarkably, short-term slice-culture-generated CD69^HI^CD4^+^ T cells phenotypically resembled *ex vivo* intrahepatic CD69^HI^CD4^+^ T cells, with high expression of CXCR6, CD49a, CCR5 and PD-1, low expression of S1PR1 and a largely resting (KLRG-1^−^CD127^+^) phenotype (figure 6E–F). Correspondingly, CD69^INT^CD4^+^ T cells also generated through hepatic slice culture acquired many of the phenotypical characteristics of their *ex vivo* counterparts, notably expression of CXCR3, and partial acquisition of the residency markers CD49a and CXCR6. Together these results suggest that CD4^+^ T cell contact with hepatic epithelia promotes their differentiation to a CD69^INT^CD4^+^ phenotype in the liver, whereas the generation of CD69^HI^CD4^+^ T_RM_ requires additional signals from the liver microenvironment.

## Discussion

In this study, we characterised two distinct CD69-expressing CD4^+^ T-cell populations in the human liver — a long-lived CD69^HI^CD4^+^ T_RM_ subset, with a prototypical tissue-retention signature, a resting restrained phenotype and the ability to instigate type-1 multifunctional responses on stimulation; and a novel population of CD69^INT^CD4^+^ T cells with a CXCR3^+^CXCR1^+^CX_3_CR1^+^ phenotype that are more activated, recirculation-competent and skewed towards T_H_2 responses on stimulation. We show that these two populations possess different generation requirements and are equipped to play differential roles in liver disease.

In agreement with other human CD4^+^ T_RM_ studies, liver CD69^HI^CD4^+^ T cells expressed T_RM_-associated retention molecules CD49a and CXCR6,^8 16 39^ have low expression of the homing receptors S1PR1 and CX_3_CR1,^13^ a resting and restrained phenotype including high PD-1 expression,^13 39^ and the ability to produce T_H_1 cytokines.^33 34 39^ CXCR6 is of particular importance as a key liver retention molecule that is required for residence of multiple lymphocyte subpopulations in the liver,^40–42^ and our data demonstrate that the liver microenvironment is able to rapidly induce a CXCR6^+^ signature in newly formed resident CD4^+^ T cells.

We recently described human liver CD8^+^ T_RM_ that share some of these key features (CXCR6^+^, PD-1^+^ and rapid functionality).^5^ Intriguingly, intrahepatic CD8^+^ T_RM_ in both mouse and humans are thought to uniquely reside within the liver vasculature.^5 43 44^ Our data suggest that liver CD69^HI^CD4^+^ T_RM_ can also be found throughout the parenchyma, including within sinusoids. Candidate molecules for maintaining the CD69^HI^T_RM_ in this niche include CXCR6 through interactions with its ligand CXCL16, expressed on the sinusoidal lumen,^23 40^ or integrin αLβ2-ICAM interactions.^44^ Furthermore, our findings suggest CD69^HI^CD4^+^T_RM_ can be found in portal regions, likely directed specifically to portal vasculature by CCR5 ligands.^45^ The strategic positioning of CD4^+^ T_RM_ in both vascular sites could allow efficient targeted immunosurveillance and opportunities to interact with other key immune cells within the liver. Finally, we demonstrated our CD69^HI^CD4^+^T_RM_ are most likely enriched for IL-21^+^T_FH_-like cells, in keeping with the emerging overlap between T_FH_ and T_RM_ phenotypes.^46 47^

Hepatic CD69^INT^CD4^+^ T cells have not been previously described. Functionally, CD69^INT^CD4^+^ T cells were most able to produce the T_H_2 cytokine IL-4. Key distinguishing features of hepatic CD69^INT^CD4^+^ T cells were expression of CXCR3 and CXCR1 required for hepatocyte homing,^21^ and importantly, CX_3_CR1. CD69^INT^CD4^+^ T cells were also found in hLNs, consistent with a wide-ranging immune surveillance role, analogous to the CX_3_CR1^INT^ ‘peripheral memory’ CD8^+^ T cells that survey peripheral tissues in both humans and mice.^19 20^ Further, a small population of non-resident CD69^INT^CD8^+^ T cells in mice has been previously reported.^48^ The ‘migratory memory’ CD4^+^ T cells described by Watanabe *et al* showed variable CD69 positivity and recirculated through the skin slower than conventional T_CM_.^49^ Interestingly, activated, recirculating CD69^INT^CD4^+^ T cells, rather than resident CD69^HI^CD4^+^ T cells correlated with necroinflammatory scores in CHB. This suggests CD69^INT^CD4^+^ T-cell involvement in proinflammatory processes in chronic viral disease, but whether this contributes to the cause, or is a consequence of the inflammation, remains to be determined.

Although CD69^INT^CD4^+^ T cells expressed a tissue egress signature (S1PR1 and CX_3_CR1) and were found in the blood, these cells may also contain a population capable of long-term residence. While this resident pool could conceivably derive from resident CD69^HI^CD4^+^ T cells, the clear disparity in chemokine receptor expression suggests this outcome is unlikely. Resident CD69^HI^CD4^+^ T cells may use CXCR6 for long-term retention more than CD69^INT^CD4^+^ T cells, allowing the possibility of distinct liver immunosurveillance roles. Alternatively, short-term resident populations may exist within the CD69^INT^CD4^+^ pool, as has been described for murine tissue CD4^+^ T cells,^50^ possibly with the potential to transdifferentiate into long-term ‘conventional’ CD69^HI^T_RM_ or ‘alternative’ CD69^INT^T_RM_. Future studies using single-cell sequencing approaches or TCR-repertoire analysis to further dissect the CD69^INT^CD4^+^ T-cell compartment are necessary to address these hypotheses.

Our data revealed insights into the mechanisms behind the generation of both CD69^INT^CD4^+^ and CD69^HI^CD4^+^ T cells. CD69^INT^CD4^+^-like cells were generated following short-term direct contact with hepatic epithelial cell lines, and primary BEC. Although the molecular mechanism for this remains undefined, *in situ* hepatocyte contact may promote CD4^+^ T-cell CD69 upregulation to an intermediate level, increasing liver dwell time and allowing more efficient immunosurveillance. Conversely, CD69^HI^CD4^+^ T cells required additional signals from the liver microenvironment, given that cells with this phenotype could only be formed from blood-derived CD4^+^ T cells when cocultured with autologous liver slices. Interestingly, robust initiation of a residency transcriptional programme can happen within 2 days in mice.^48^ Cytokines such as IL-15 and TGF-β can induce a CD8^+^ T-cell tissue residency programme,^11^ and we previously demonstrated that combinations of both cytokines were sufficient to generate cells with a CD8^+^ T_RM_ phenotype.^5^ This raises the possibility that these cytokines provide the same additional signals for CD69^HI^CD4^+^T_RM_ formation. Unfortunately, extension of liver-slice coculture beyond 5 hours was not technically possible. Mouse models could be used to better test the longevity of these phenotypes.

In conclusion, this study provides a phenotypical and functional framework for understanding liver CD4^+^ T_RM_ biology and characterises a novel heterogeneous CD69^INT^CD4^+^ T-cell population that is shaped by the liver microenvironment. We suggest that for at least some peripheral tissues, binary expression of CD69 alone is not sufficient to define tissue-resident CD4^+^ T cells. This work will facilitate the understanding of the role of liver CD4^+^ T cells in hepatic immune homeostasis, with implications for the development of novel immunotherapeutic strategies for chronic liver diseases.

10.1136/gutjnl-2020-323771.supp10 Supplementary data

## Data Availability

Data are available upon reasonable request.
