# Supplementary material for: The human liver microenvironment shapes the homing and function of CD4+ T-cell populations
Source: Gut. 2021 Sep 21;71(7):1399–411. doi: 10.1136/gutjnl-2020-323771 (PMC9185819; doi:10.1136/gutjnl-2020-323771)
Supplement: Supplementary data [file gutjnl-2020-323771supp011.pdf]

Supplementary information:

Supplementary Experimental Procedures

Immune cell isolation

Peripheral blood mononuclear cells (PBMCs) were isolated from human blood by density centrifugation (Lympholyte-H [Cederlane] – centre A, Ficoll-Hypaque plus [GEHealthcare] – centre B). Intrahepatic lymphocytes (IHL) were isolated through either chopping, multiple PBS washes, digestion with a stomacher machine (Seward), filtering out debris and density centrifugation (centre A); or for centre B biopsies – mechanical disruption with cell scrapers, filtration to remove debris, centrifugation. Larger centre B resections and explants: enzymatic digestion (30min incubation at 37°C with 0.02% Collagenase IV [Thermo-Fisher] 0.002% DNase I [Sigma-Aldrich]), mechanical disruption through a GentleMACS dissociator (Miltenyi Biotec), then 70μm filtering and centrifugation as above. Lymph node and spleen mononuclear cells were isolated through manual dissection, GentleMACS dissociation, filtration, and density centrifugation on Lympholyte-H (centre A); or manual dissection, filtration, and centrifugation on a Pancoll (PanBiotech) gradient. Intraepithelial lymphocytes (IEL) were isolated from the duodenum and Ileum of the gut by enzymatic digestion (1hr incubation at 37°C shaking [180rpm] with 0.15% Collagenase IV [Thermo-Fisher] 0.02% DNase I [Sigma-Aldrich] 0.0025% Hyaluronidase Type IV-S [Sigma-Aldrich] 0.00125% Liberase DL [Roche]), mechanical disruption using a syringe, then 70μm filtration and centrifugation on a Pancoll gradient as above. The isolation of IHLs from FNA samples was undertaken as previously described[35]. In brief, samples were centrifuged; the remaining cell pellet was re-suspended in 2mL of red blood cell lysis buffer (Biolegend) for 5min on ice, prior to staining. All samples were used immediately. Any samples for later use were frozen in 10% DMSO (Sigma-Aldrich) in fetal bovine serum (FBS) and stored in accordance with the Human Tissue Act.

Antibodies for Immunofluorescence

The following antibodies were used: mouse anti-human CD4 (Novus Biologicals; NBP2-46149), mouse anti-human NKp46 (R&D systems, UK; MAB1850), Rabbit anti-human CX3CR1 (Thermofisher, UK; 702321) and rabbit-human CXCR6 (Abcam, UK; ab8023). Single stains and IMCs were used to ensure specificity. Secondary antibodies were all purchased from Thermo-Fisher Scientific.

T cell culture for stimulation experiments

PBMCs/IHLs were pre-stained with antibodies against CD69, CD4, CD56, and γδ-TCR (centre A only), washed twice in PBS, and plated out in 96-well plates at 10^6 cells/well in T-cell media (RPMI [ThermoFisher] + 10% FBS (Sigma-Aldrich), 100U/ml penicillin, 100μg/ml streptomycin, 1% non-essential amino acids (NEAA), 1% L-glutamine [all ThermoFisher Scientific, UK]) containing T cell stimulants.

T cell co-culture experiments

Hepatic cell lines all cultured in 24-well plates in 1ml media as follows: Huh-7, HepG2, Hep3B – all in complete DMEM (ThermoFisher): DMEM + 10% FBS, 100U/ml penicillin,

100μg/ml streptomycin, 1% NEAA, 1% L-glutamine; LX-2 – as above but 2% FBS; primary BEC – 1:1 Ham's F12 media (ThermoFisher) & DMEM + 10% heat activated human serum (HD supplies) 2mM Penicillin/Streptomycin, 10μg/L epidermal growth factor, 10μg/L hepatocyte growth factor (both Peprotech), 124 IU/L Insulin, 20μg/L Hydrocortisone (both QE hospital pharmacy, Birmingham), 10μg/L Cholera Toxin, 0.2nM Tri-iodothyronine (both Sigma-Aldrich); primary HSEC – Human Endothelial Serum free media (ThermoFisher) + 10% heat activated human serum, 10μg/L hepatocyte growth factor, 10μg/L vascular endothelial cell growth factor (Peprotech). BEC and HSEC cultured on type-1 rat-tail collagen (Sigma Aldrich, UK) coated plates (coated with 40μg/ml solution). Once adhered to plate, T cells added to 1ml wells in 100μl of T-cell media.

Supplementary Figures:

Supplementary Figure 1 – Gating strategies used to identify and compare hepatic $CD69^-$, $CD69^{INT}$ and $CD69^{HI}$ cells. Gating strategy used in centre A (**A**), and B (**B**) where blue arrows represent direction of gating (onward gate in bold when multiple in one plot).

Supplementary Figure 2 – Homing, location, and naïve/memory profiles of CD69-deliniated subsets. A – Comparison of each subset (%) recovered from liver tissue digests or fine needle aspirate (FNA) samples. **B** – proportion of liver $CD4^+$ T cells in each subset that comprise naïve, central memory (T_{CM}), effector memory (T_{EM}), or T_{EMRA} as shown in representative plots and combined stack chart (n=51). **C –** Expression of additional homing/retention molecules in each subset. **D -** Immunofluorescent staining of formaldehyde-fixed paraffin-embedded liver sections (from patient with PBC). White arrows indicate CX_3CR1^+ $CD4^+$ T cells. Statistical comparisons by Wilcoxon matched-pairs signed rank tests (part A); Freidman tests with Dunn's multiple tests for parts B-C.

Supplementary Figure 3 - Localisation of CXCR6- and CX_3CR1-expressing $CD4^+$T-cells throughout the liver. A – Example stains for each chemokine receptor merged and split by channel. **B –** Presence of cells of interest in central areas (**D**), and fibrotic areas (**E**). Central areas n=14 (5 donor, 4 HBV, 5 PBC), fibrotic areas n=9 (4 HBV, 5 PBC). Areas of interest a-c show higher magnification of cells of interest (white arrows – cell of interest, green arrows – $NKp46^+$ cell, yellow arrow – chemokine receptor$^+$ $CD4^-$ cell. Yellow scale bars - 50μm, white scale bars – 20μm.

Supplementary Figure 4 – Conventional regulatory T cells are not enriched in any CD69-designated subset. A – Representative gating for T_{REGS} ($CD25^{HI}CD127^{LO}$) in each subset. Violin plot shows combined expression data. **B** – Expression of T_{REG} cardinal features in each subset – CTLA-4 and CD39 by representative staining and combined total data. Freidman tests with Dunn's multiple tests used for statistical comparison throughout.

Supplementary Figure 5 – Differential cytokine responses of intrahepatic subsets marked by CD69 expression. A – Cytokine expression following 5-hour stimulation of IHL with PMA and Ionomycin, followed by intracellular staining. IL-2 (n=17), TNF-α (n=16), IFN-γ (n=19), IL-4 (n=18), IL-17 (n=12), IL-10 (n=17), IL-21 (n=11). **B –** Expression of cytokines following 5hr in absence of stimulation, followed by intracellular staining. IL-2 (n=19), TNF-α (n=18), IFN-γ (n=21), IL-4 (n=20), IL-17 (n=11), IL-10 (n=19), IL-21 (n=13). **C -** Multi-functional responses of liver $CD69^-$, $CD69^{INT}$, and $CD69^{HI}$ $CD4^+$ T cells following 5-hour PMA/Ionomycin stimulation. Stacked bar chart heights represent median % of each combination of IL-2/TNF-α/IFN-γ-expressing cells as shown (n=7). Table displays statistical significance between the

Supplemental material BMJ Publishing Group Limited (BMJ) disclaims all liability and responsibility arising from any reliance placed on this supplemental material which has been supplied by the author(s) Gut

three populations, and interquartile range values. Freidman tests with Dunn's multiple tests used to compare matched populations throughout.

Supplementary Figure 6 – Transcription Factor profiles of Liver CD4+ T cell subsets. Representative plots and combined % expression of T-bet (n=19), GATA-3, Rorγt and FoxP3 (all n=11) amongst CD69-designated subsets. Freidman tests with Dunn's multiple tests used for statistical comparisons.

Supplementary Figure 7 – Additional correlation analysis with clinical parameters in HBV patient livers. A – Frequency of each subset in Hepatitis B e antigen positive vs –negative patients (Mann Whitney Test). **B –** Correlation of patient serum ALT against frequency of each subset. Spearman's correlation analysis p and r values given. **C –** Frequency of each subset in chronic HBV patient donor livers at each HBV disease stage. Disease staging of HBV donors determined by combination of HBeAg, HBV DNA, and serum ALT (compared by Kruskal-Wallis tests with multiple Dunn's post-hoc testing). Only 1 donor was at immunotolerant stage, so this stage excluded from dataset shown.

Supplementary Figure 8 – CD69^{INT} generation occurs with primary human epithelia and is contact-dependent. CD69 expression in peripheral blood-derived CD4+ T cells following co-culture for 1,3, and 7 days alone, with Huh-7 cells, or BEC. Data displayed as representative flow cytometry plots (**A**), and combined donor data alongside HSEC and LX-2 culture conditions (**B**)**.** Median + 95% CI shown (n=3). **C –** CD69% expression amongst PBMC-derived CD4+ T cells when either cultured overnight with Huh-7 cells directly (direct contact), or when separated by a 0.4µm pore transwell insert (indirect contact). **D –** Naïve/Memory CD4+ T cell purity following isolation, followed by % CD69 expression following 16h co-culture with Huh-7 cells (n=1, 2 technical replicates).

Supplementary Figure 9 – Co-culture with hepatic epithelia infers CD4+ T cells with IL-4 production capacity. CD4+ T cells isolated from healthy human blood were cultured for 5 hours either alone or with Huh-7 hepatic epithelial cells, harvested and stimulated for 4 hours with anti-CD3/CD28 or PMA/Ionomycin, and then assessed for their ability to produce prototypic T cell cytokines by intracellular staining and flow cytometry. **A –** Staining example for IL-4 in T cells alone and following co-culture (PMA/Ionomycin stimulation). **B –** % expression of all the cytokines studied in co-cultured cells as a fold change from control (T cell only) cells (anti-CD3/CD28 – top, PMA/Ionomycin stimulation – bottom). **C –** Percentage expression of IL-4 in co-cultured CD69+ versus co-cultured CD69- cells (both stimulation methods shown). Data from this experiment compiled from three independent donors.

Wiggins BG, *et al. Gut* 2022; 71:1399–1411. doi: 10.1136/gutjnl-2020-323771

Supplementary Tables:

Supplementary Table 1 – Patient information for Liver tissue donors.
Shown are the disease breakdowns, sample type acquired and donor numbers in each group, together with median and IQR of the ages, and gender in each group. Numbers of donors acquired from each centre (A – University of Birmingham, B – University College London). AIH – Autoimmune hepatitis, ALD – Alcoholic liver disease, ARLD – Alcohol-related liver disease, BCM – breast cancer metastasis, CRC – colorectal cancer, HBV – hepatitis B virus, HCC – hepatocellular carcinoma, HCV – hepatitis C virus, MM – melanoma metastasis, NASH – non-alcoholic steatohepatitis, PBC – Primary biliary cholangitis, PSC – primary sclerosing cholangitis, NCPH – non-cirrhotic portal hypertension, SBC – secondary biliary cholangitis. * - 2 patient ages missing, ** - 3 patient ages missing, *** - 6 patient ages missing, † - 2 patient genders missing, †† - 4 patient genders missing, ††† - 6 patient genders missing from available data.

Aetiology	Sample Type	Numbers	Median age (IQR)	% Female	Centre A/B
Control	Donor Explant	12	51 (22)*	25††	A(8) B(4)
Control	Pre-implant donor biopsy	7	39* (14)	33††	B
Control	CRC margin	45	61 (18)***	20††	B
Control	HCC margin	11	62.5 (8.8)**	25†	B
Control	BCM margin	1	57	100	B
Control	MM margin	1	83	0	B
Control	PLD	3	61 (7)	100	A
HBV	Explant	4	39 (3)*	50†	A(1) B(3)
HBV	Biopsy	51	35 (17.25)	29†††	B
ALD	Explant	20	59 (14.3)	15	A
ARLD	Explant	1	48	0	B
NASH	Explant	8	60.5 (9.3)	50	A
NASH	Explant	1	51	0	B
PBC	Explant	7	44 (11.5)	71	A
PSC	Explant	12	35.5 (12.3)	25	A
AIH	Explant	1	21	0	A
HCV	Explant	2	59 (6)	0	A
HCV	Explant	3	61 (9)	33	B
Cryptogenic	Explant	2	53.5 (10.5)	50	A
Budd Chiari	Explant	1	31	0	A
NCPH	Explant	1	63	0	A
SBC	Explant	1	58	100	A

Supplementary Table 2 – Patient information for lymph node, gut and spleen donors

Aetiology	Sample Type	Numbers	% Female	Centre A/B
Donor	Hepatic hilar LN	6	A(4) B(2)	
Donor	Non-hepatic LN (mesenteric)	6	B	
Donor	Biopsy – Duodenum	4	B	
Donor	Biopsy – Ileum	2	B	
Donor	Spleen	4	A(1) B(2)	

Supplementary Table 3 – Antibodies used in flow cytometry experiments

Antigen	Fluorochrome	Manufacturer	Clone	Catalogue Number
**Phenotype**				
CCR10	PE	Biolegend	6588-5	341504
CCR5	PE	Biolegend	J418F1	359106
CCR6	AlexaFluor488	Biolegend	G034E3	353414
CCR7	PE-Cy7	Biolegend	G043H7	353226
CCR9	PerCP-Cy5.5	Biolegend	L053E8	358906
CD103	APC	BD Biosciences	Ber-ACT8	563883
CD103	BV605	Biolegend	Ber-ACT8	350218
CD103	FITC	Biolegend	Ber-ACT8	350203
CD127	BV510	Biolegend	A019D5	351332
CD25	PE-Cy5	Biolegend	BC96	302608
CD27	FITC	Biolegend	M-T271	356404
CD3	BV711	Biolegend	OKT3	317328
CD3	BV711	BD Biosciences	UCHT1	563546
CD38	APC-Vio770	Mltenyi Biotec	REA572	130-099-151
CD39	BV421	Biolegend	A1	328214
CD4	APC	BD Biosciences	RPA-T4	555349
CD4	BV510	Biolegend	SK3	344634
CD4	FITC	Biolegend	OKT4	317408
CD4	APC-Cy7	BD Biosciences	RPA-T4	557871
CD4	BV421	BD Biosciences	RPA-T4	562425
CD45	BUV805	BD Biosciences	HI30	564914
CD45RA	BV421	BD Biosciences	HI100	562885
CD45RA	PE-Cy7	Biolegend	HI100	3014126
CD45RA	eFluor450	Thermo Fisher	HI100	48-0458-41
CD49a	PE	Biolegend	TS2/7	328304
CD49d	BV421	Biolegend	9F10	304322
CD56	APC-Vio770	Mltenyi Biotec	REA196	130-100-690
CD56	BUV395	BD Biosciences	NCAM16.2	563555
CD69	FITC	BD Biosciences	FN50	560969
CD69	PE-Dazzle594	Biolegend	FN50	310942
CD69	BV605	Biolegend	FN50	310937
CD8	BV786	Biolegend	RPA-T8	301046
CD8	PE-Cy5	Biolegend	RPA-T8	301010
CD8	AlexaFluor700	Biolegend	RPA-T8	300518
CD8	AlexaFluor700	Thermo Fisher	OKT8	56-0086-82
CTLA-4	PE-Dazzle594	Biolegend	L3D10	349922
CX3CR1	PE-Cy7	Biolegend	2A9-1	341612
CXCR1	PE-Cy7	Biolegend	8F1/CXCR1	320620
CXCR3	AlexaFluor488	Biolegend	G025H7	353710
CXCR6	APC	Biolegend	K041E5	356005
CXCR6	PerCP-Cy5.5	Biolegend	K041E5	356010

BMJ Publishing Group Limited (BMJ) disclaims all liability and responsibility arising from any reliance placed on this supplemental material which has been supplied by the author(s)

Antigen	Fluorochrome	Manufacturer	Clone	Catalogue Number
FOXP3	PE	Thermo-Fisher	PCH101	12-4776-42
FoxP3	BV421	Biolegend	206D	320124
GATA-3	BV421	Biolegend	16E10A23	653813
GATA-3	BB700	BD Biosciences	L50-823	566643
HLA-DR	FITC	BD Biosciences	G46-6	555811
HLA-DR	BV421	Biolegend	L243	307636
HLA-DR	Horizon V500	BD Biosciences	G46-6	561224
Integrin β7	PE	Biolegend	FIB504	321204
KLRG-1	PE	Biolegend	SA231A2	367712
PD-1	PE	Biolegend	EH12.2H7	329906
RORγt	BV650	BD Biosciences	Q21-559	563424
RORγt	FITC	BD Biosciences	Q21-559	563621
S1PR1	eFluor660	Thermo Fisher	SW4GYPP	50-3639-41
T-bet	APC	Thermo-Fisher	eBio4B10	50-5825-82
γδ-TCR	APC-Vio770	Mltenyi Biotec	11F2	130-109-360
**Function**				
IFN-γ	APC	BD Biosciences	B27	554702
IFN-γ	AlexaFluor700	Biolegend	B27	506516
IFN-γ	Horizon V450	BD Biosciences	B27	560371
IL-10	BV421	Biolegend	JES3-9D7	501421
IL-10	FITC	Biolegend	JES3-9D7	501411
IL-10	PE	Biolegend	JES3-9D7	501404
IL-17A	PerCP-Cy5.5	Biolegend	BL168	512313
IL-17A	BUV605	Biolegend	BL168	512325
IL-2	PE	Thermo Fisher	MQ1-17H12	12-7029-82
IL-2	PerCP-eFluor710	Thermo Fisher	MQ1-17H12	46-70290-42
IL-2	BB700	BD Biosciences	MQ1-17H12	566406
IL-21	PE	Biolegend	3A3-N2	513004
IL-21	PE	BD Biosciences	3A3-N2.1	562042
IL-4	PE-Cy7	Biolegend	MP4-25D2	500824
IL-4	AlexaFluor647	Biolegend	MPA-25D2	500818
Ki-67	PB	Biolegend	Ki-67	350512
TGF-β	APC	Novus Biologicals	1D11	IC420A
TNF-α	eFluor450	Thermo Fisher	MAb11	48-7349-42
TNF-α	FITC	BD Biosciences	MAb11	554512
TNF-α	PE-Cy7	Biolegend	Mb11	502930
**HLA-haplotyping**				
HLA-A2	FITC	BioRad	BB7.2	MCA2090
HLA-A2	PE-Cy7	Biolegend	BB7.2	343302
HLA-A3	PE	Thermo Fisher	GAP.A3	11-5754-42
HLA-A9	APC	Miltenyi Biotec	Rea127	130-099-540

Wiggins BG, *et al. Gut* 2022; 71:1399–1411. doi: 10.1136/gutjnl-2020-323771

Supplementary Table 4 – Disease aetiology for functional experiments

Figure	Cytokine	Condition	n=	Breakdown
4A	IL-2	Anti-CD3/CD28	27	2 PBC, 5 control donor, 12 CRC, 1 ARLD, 1 BCM, 1 PSC, 1 ALD, 1 NASH, 1 PLD, 1 Cryptogenic, 1 Budd Chiari
4B	TNF-α	Anti-CD3/CD28	24	2 PBC, 5 control donor, 11 CRC, 1 PSC, 1 ALD, 1 NASH, 1 PLD 1 Cryptogenic, 1 Budd Chiari
4C	IFN-γ	Anti-CD3/CD28	24	2 PBC, 5 control donor, 11 CRC, 1 PSC, 1 ALD, 1 NASH, 1 PLD 1 Cryptogenic, 1 Budd Chiari
4D	IL-4	Anti-CD3/CD28	18	2 PBC, 9 CRC, 1 PSC, 1 ALD, 1 ARLD, 1 BCM, 1 NASH, 1 PLD, 1 Budd Chiari
4E	IL-17	Anti-CD3/CD28	16	2 PBC, 5 control donor, 3 CRC, 1 BCM, 1 ARLD, 1 PLD, 1 cryptogenic, 1 NASH, 1 Budd Chiari
4F	IL-10	Anti-CD3/CD28	22	2 PBC, 1 NASH, 1 PLD, 1 Budd Chiari, 1 PSC, 5 control donor, 9 CRC, 1 BCM, 1 ARLD
4G	IL-21	Anti-CD3/28	11	9 CRC, 1 ARLD, 1 BCM
S5A	IL-2	PMA/Ionomycin	17	2 PBC, 1 PSC, 1 ALD, 1 ARLD, 7 CRC, 1 BCM, 1 NASH, 1 PLD 1 Cryptogenic, 1 Budd Chiari
S5A	TNF-α	PMA/Ionomycin	16	2 PBC, 2 control donor, 6 CRC, 1 PSC, 1 ALD, 1 NASH, 1 PLD 1 Cryptogenic, 1 Budd Chiari
S5A	IFN-γ	PMA/Ionomycin	19	2 PBC, 2 control donor, 1 PSC, 1 ALD, 1 NASH, 1 PLD 1 Cryptogenic, 1 Budd Chiari, 1 ARLD, 7 CRC, 1 BCM
S5A	IL-4	PMA/Ionomycin	18	2 PBC, 2 control donor, 1 PSC, 1 ALD, 1 NASH, 1 PLD 1 Cryptogenic, 1 Budd Chiari, 1 ARLD, 7 CRC, 1 BCM
S5A	IL-17	PMA/Ionomycin	12	2 PBC, 2 control donor, 1 ALD, 1 NASH, 1 PLD 1 Cryptogenic, 1 Budd Chiari, 1 ARLD, 1 CRC, 1 BCM
S5A	IL-10	PMA/Ionomycin	17	2 PBC, 2 control donor, 1 PSC, 1 NASH, 1 PLD, 1 Budd Chiari, 1 ARLD, 7 CRC, 1 BCM
S5A	IL-21	PMA/Ionomycin	11	2 control donors, 7 CRC, 1 ARLD, 1 BCM
S5B	IL-2	Unstimulated	19	2 PBC, 1 PSC, 1 ALD, 1 ARLD, 9 CRC, 1 BCM, 1 NASH, 1 PLD 1 Cryptogenic, 1 Budd Chiari
S5B	TNF-α	Unstimulated	18	2 PBC, 2 control donor, 1 PSC, 1 ALD, 9 CRC, 1 NASH, 1 PLD 1 Cryptogenic, 1 Budd Chiari
S5B	IFN-γ	Unstimulated	21	2 PBC, 2 control donor, 1 PSC, 1 ALD, 1 ARLD, 9 CRC, 1 BCM, 1 NASH, 1 PLD 1 Cryptogenic, 1 Budd Chiari
S5B	IL-4	Unstimulated	20	2 PBC, 2 control donor, 1 PSC, 1 ALD, 1 ARLD, 9 CRC, 1 BCM, 1 NASH, 1 PLD, 1 Budd Chiari
S5B	IL-17	Unstimulated	11	2 PBC, 8 CRC, 2 control donor, 1 ALD, 1 NASH, 1 PLD, 1 Budd Chiari, 1 cryptogenic,
S5B	IL-10	Unstimulated	19	2 PBC, 2 control donor, 1 PSC, 1 ARLD, 9 CRC, 1 BCM, 1 NASH, 1 PLD, 1 Budd Chiari
S5B	IL-21	Unstimulated	13	2 control donors, 9 CRC, 1 ARLD, 1 BCM
S5C	IL-2/TNF-α/IFN-γ	PMA/Ionomycin	7	2 PBC, 1 PSC, 1 ALD, 1 NASH, 1 PLD, 1 Budd Chiari

Wiggins BG, *et al. Gut* 2022; 71:1399–1411. doi: 10.1136/gutjnl-2020-323771
