# Supplementary material for: The human liver microenvironment shapes the homing and function of CD4+ T-cell populations
Source: Gut. 2021 Sep 21;71(7):1399–411. doi: 10.1136/gutjnl-2020-323771 (PMC9185819; doi:10.1136/gutjnl-2020-323771)

Supplementary Figure 1 – Gating strategies used to identify and compare hepatic CD69^{-}, CD69^{INT} and CD69^{HI} cells.

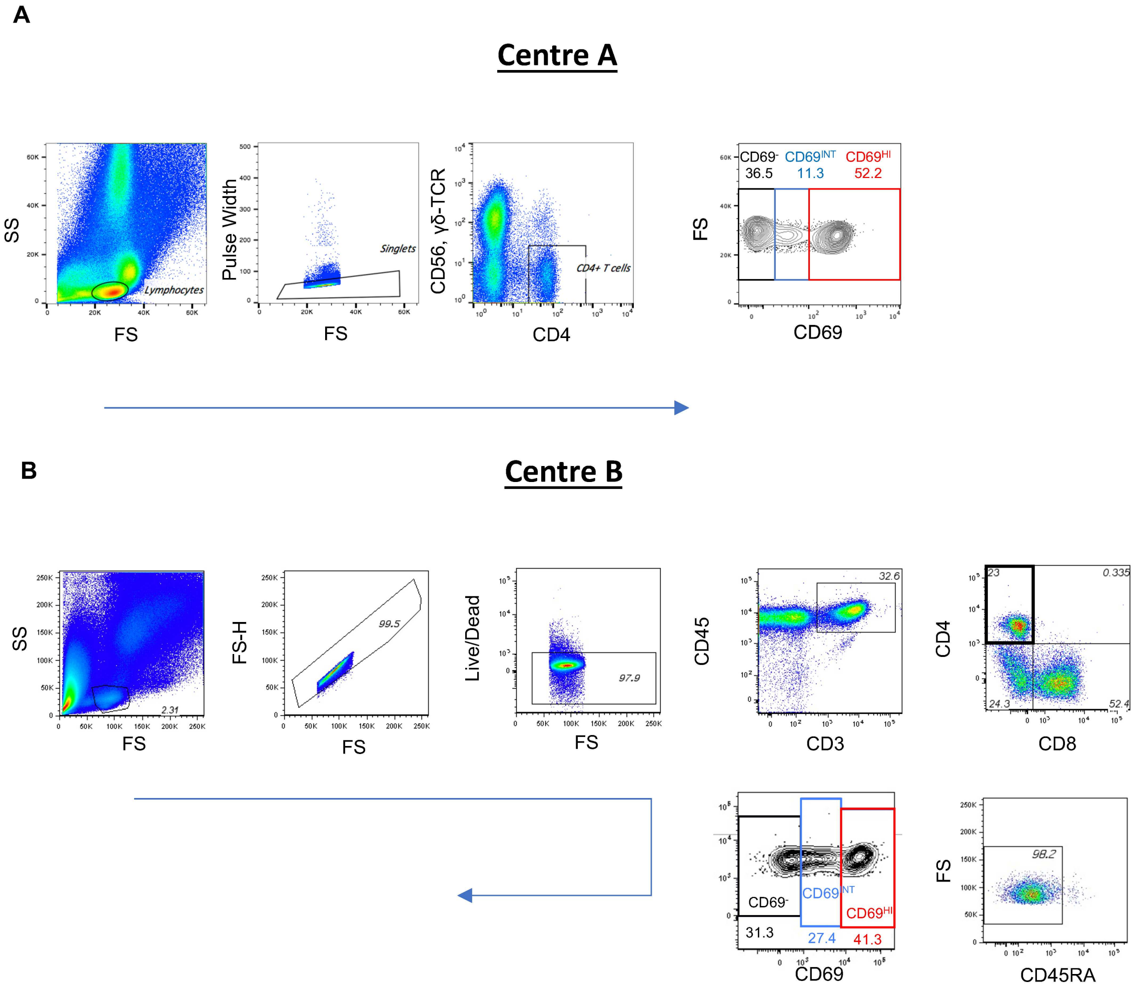

Supplement: Supplementary data [file gutjnl-2020-323771supp001.pdf]
