# Supplementary figures and images for: The human liver microenvironment shapes the homing and function of CD4+ T-cell populations
Source: Gut. 2021 Sep 21;71(7):1399–411. doi: 10.1136/gutjnl-2020-323771 (PMC9185819; doi:10.1136/gutjnl-2020-323771)

Supplementary Figure 2 – Homing, location, and naïve/memory profiles of CD69-deliniated subsets.

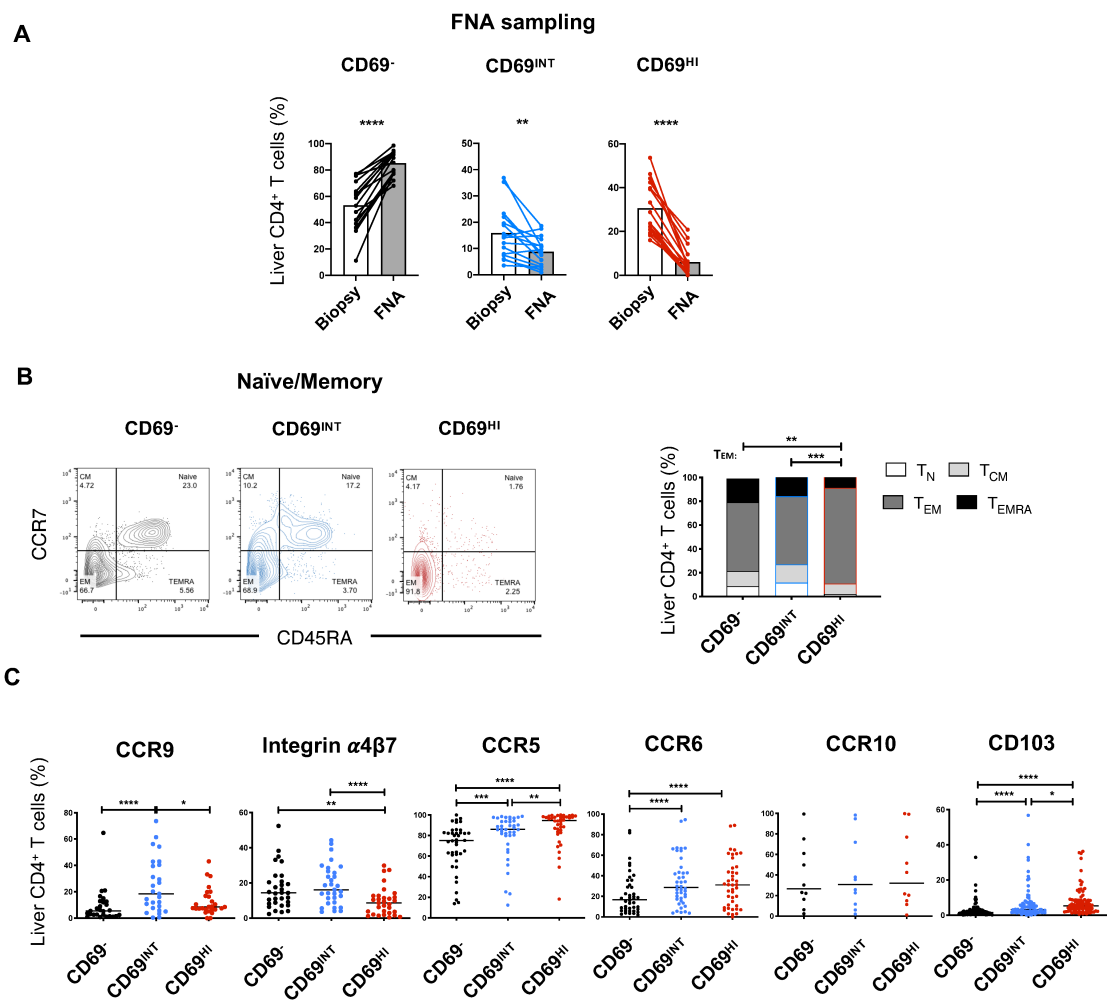

Supplement: Supplementary data [file gutjnl-2020-323771supp002.pdf]

Supplementary Figure 6 – Transcription Factor profiles of Liver CD4<sup>+</sup> T cell subsets

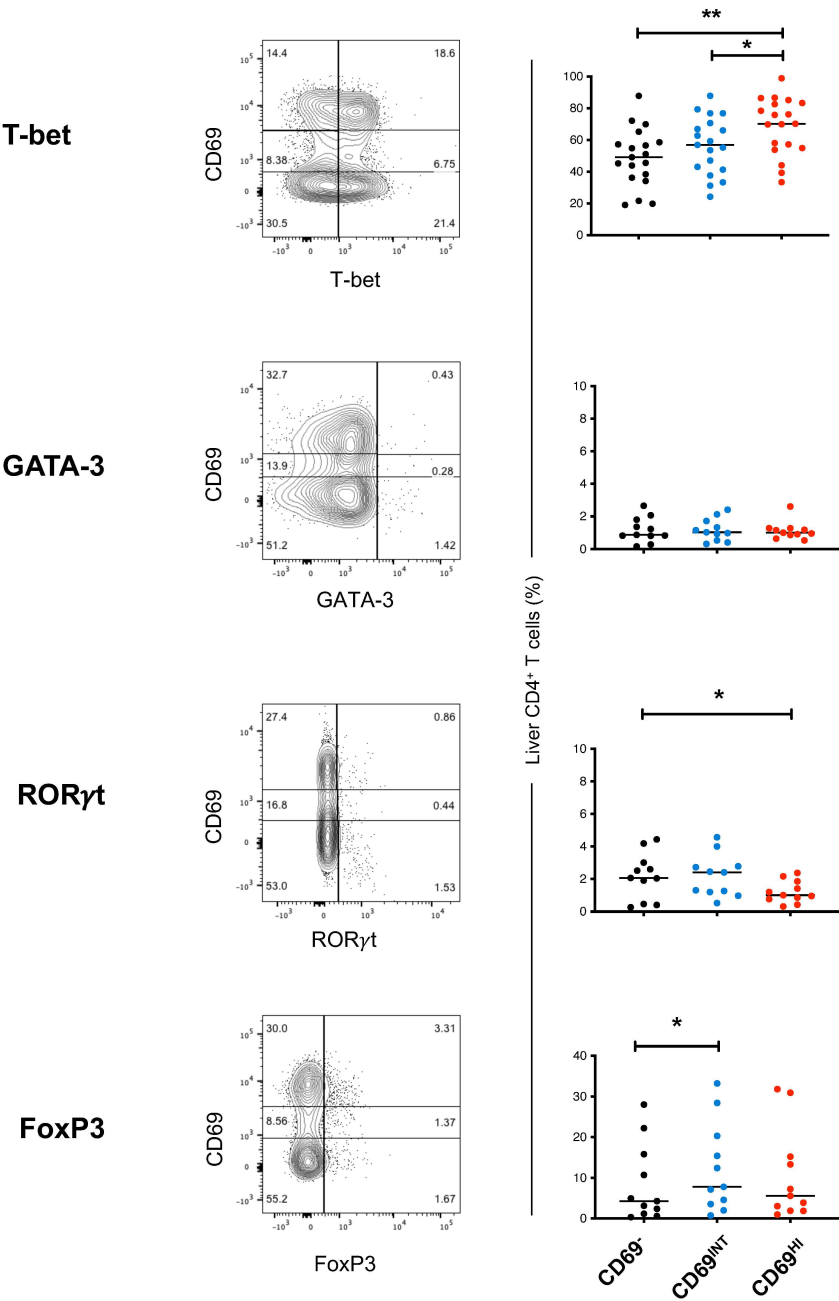

Supplement: Supplementary data [file gutjnl-2020-323771supp006.pdf]

Graphical Abstract:

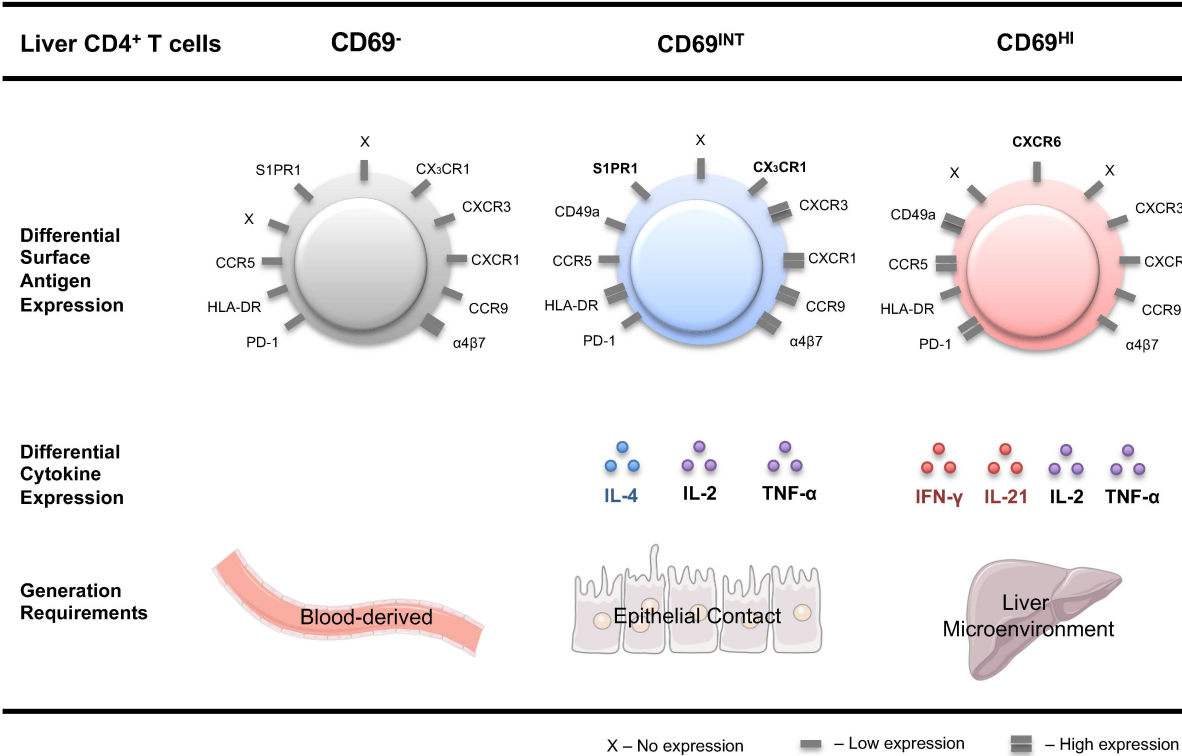

Supplement: Supplementary data [file gutjnl-2020-323771supp010.pdf]
