# Supplementary material for: The human liver microenvironment shapes the homing and function of CD4+ T-cell populations
Source: Gut. 2021 Sep 21;71(7):1399–411. doi: 10.1136/gutjnl-2020-323771 (PMC9185819; doi:10.1136/gutjnl-2020-323771)

Supplementary Figure 3 - Localisation of $CXCR6$- and CX_3CR1-expressing $CD4^+$T-cells throughout the liver

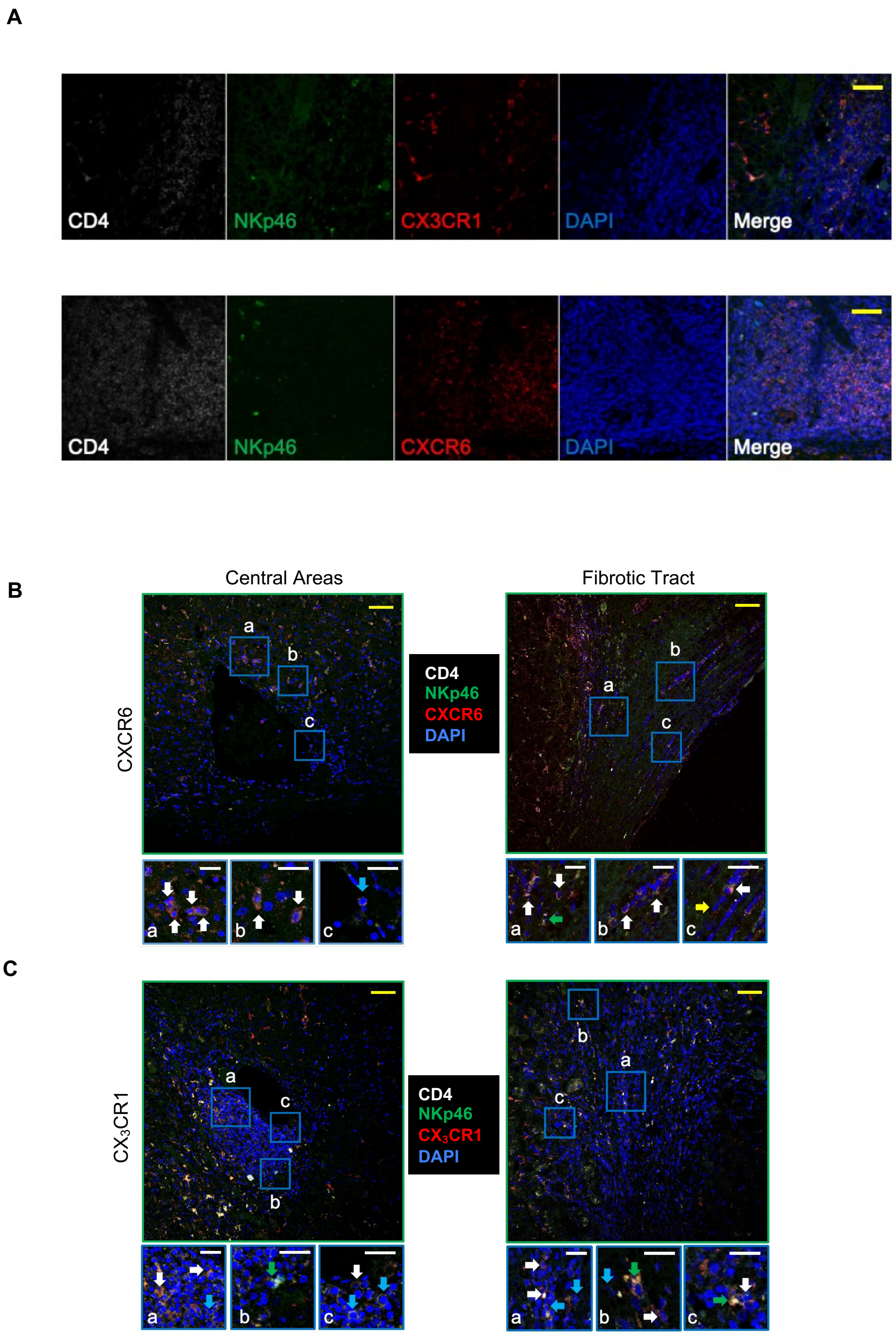

Supplement: Supplementary data [file gutjnl-2020-323771supp003.pdf]
