# Supplementary material for: The human liver microenvironment shapes the homing and function of CD4+ T-cell populations
Source: Gut. 2021 Sep 21;71(7):1399–411. doi: 10.1136/gutjnl-2020-323771 (PMC9185819; doi:10.1136/gutjnl-2020-323771)
Supplement: Supplementary data [file gutjnl-2020-323771supp005.pdf]

Supplementary Figure 5 – Differential cytokine responses of intrahepatic subsets marked by CD69 expression.

A **PMA/Ionomycin**

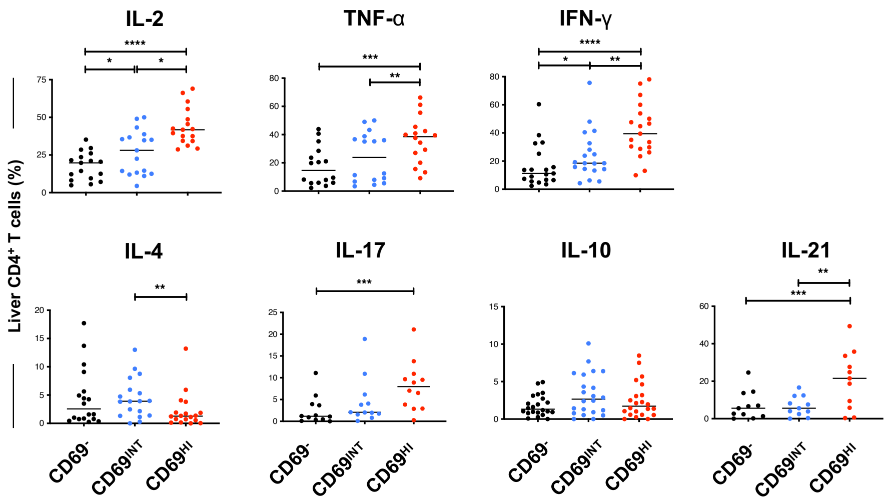

B **Unstimulated**

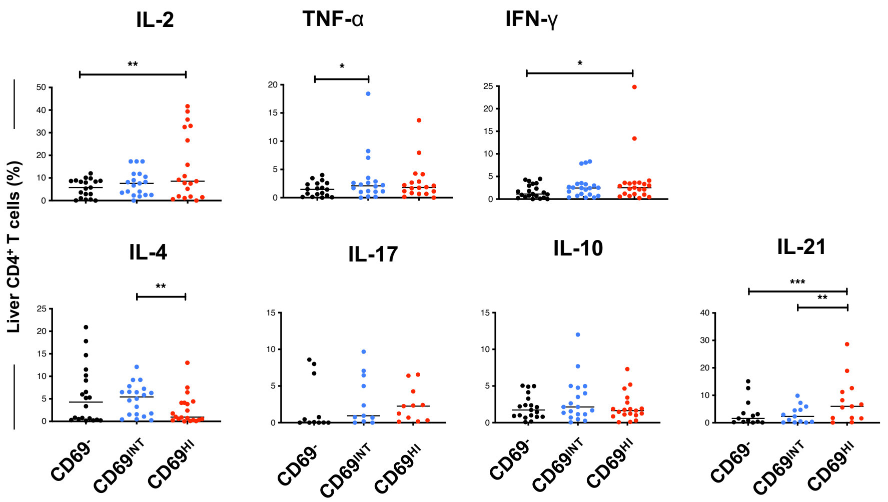

C **PMA/Ionomycin**

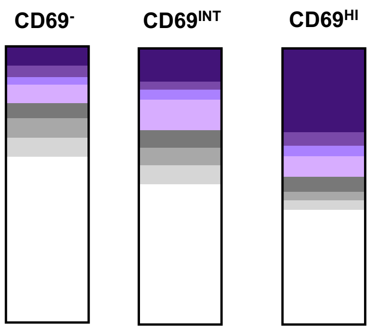

Population	Key	Significance			IQR (%)		
		- vs. INT	- vs. HI	INT vs. HI	-	INT	HI
IL-2$^+$TNF-α$^+$IFN-γ$^+$		ns	***	ns	1.1	4.5	15
TNF-α$^+$IFN-γ$^+$		ns	ns	ns	1.4	1.7	3.3
IL-2$^+$IFN-γ$^+$		ns	*	ns	1.7	2.7	2.8
IL-2$^+$TNF-α$^+$		ns	ns	ns	4.4	8.5	4.6
IFN-γ$^+$		ns	ns	ns	4.3	2.6	3.9
TNF-α$^+$		ns	**	ns	2.7	3.6	1.7
IL-2$^+$		ns	ns	ns	4.2	4.7	2.3
IL-2$^-$TNF-α$^-$IFN-γ$^-$		ns	*	ns	15	26	16
