# Supplementary material for: The human liver microenvironment shapes the homing and function of CD4+ T-cell populations
Source: Gut. 2021 Sep 21;71(7):1399–411. doi: 10.1136/gutjnl-2020-323771 (PMC9185819; doi:10.1136/gutjnl-2020-323771)

Supplementary Figure 9 – Co-culture with hepatic epithelia infers CD4⁺ T cells with IL-4 production capacity.

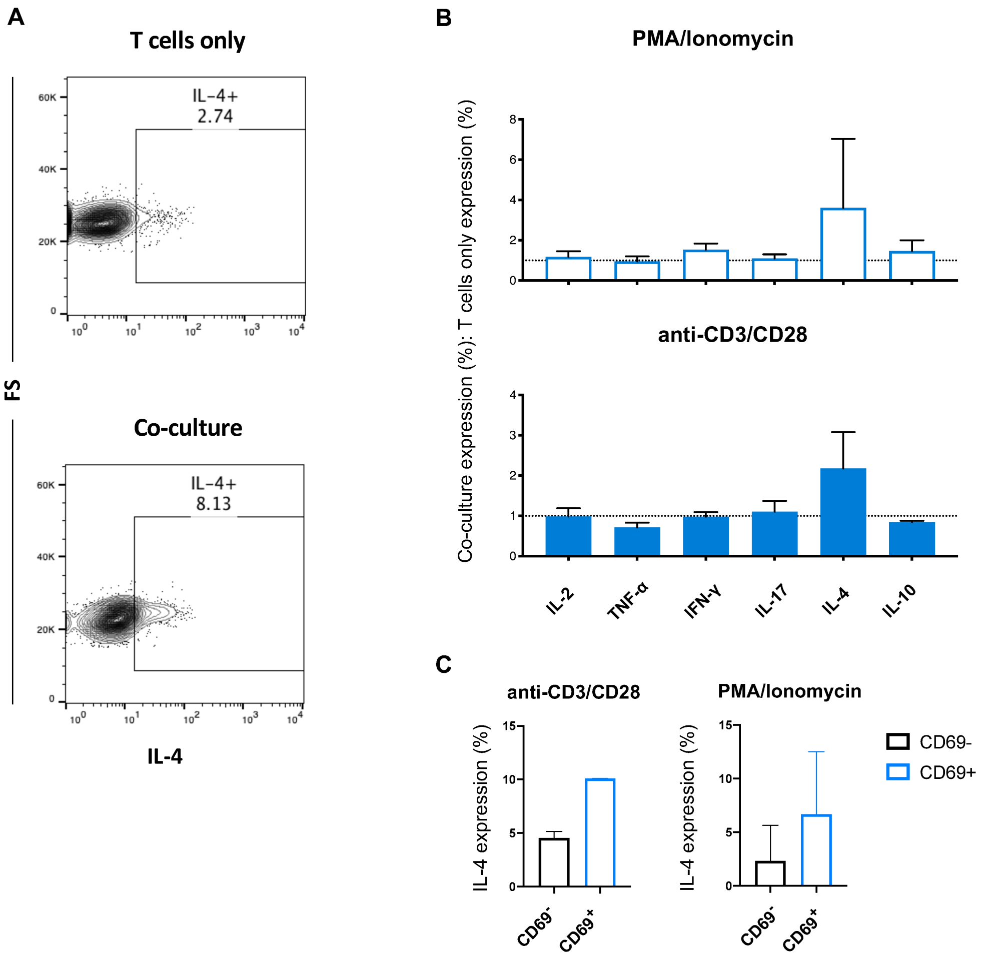

Supplement: Supplementary data [file gutjnl-2020-323771supp009.pdf]
